# Crystal structures of {1,1,1-tris­[(salicylaldimino)­meth­yl]ethane}­gallium as both a pyridine solvate and an aceto­nitrile 0.75-solvate and {1,1,1-tris[(salicylaldimino)­meth­yl]ethane}­indium di­chloro­methane solvate

**DOI:** 10.1107/S2056989020004375

**Published:** 2020-04-03

**Authors:** Dominic L. Ventura, William W. Brennessel, William S. Durfee

**Affiliations:** a Department of Chemistry, D’Youville College, 320 Porter Avenue, Buffalo, NY 14201, USA; b Department of Chemistry, University of Rochester, 120 Trustee Road, Rochester, NY 14627, USA; c Department of Chemistry, Buffalo State College, 1300 Elmwood Avenue, Buffalo, NY 14222, USA

**Keywords:** crystal structure, gallium, indium, sexadentate ligand, infra-red spectroscopy, IR, NMR

## Abstract

The sexa­dentate ligand 1,1,1-tris­[(salicyl­idene­amino)­meth­yl]ethane has been reported numerous times in its triply deprotonated form coordinated to transition metals and lanthanides, yet it has been rarely employed with main-group elements, including in substituted forms. Its structures with gallium and indium are reported as solvates, namely, ({[(2,2-bis­{[(2-oxido­benzyl­idene)amino-κ^2^ *N*,*O*]meth­yl}prop­yl)imino]­meth­yl}phenololato-κ^2^ *N*,*O*)gallium(III) pyridine monosolvate, [Ga(C_26_H_24_N_3_O3)]·C_5_H_5_N, the aceto­nitrile 0.75-solvate, [Ga(C_26_H_24_N_3_O_3_)]·0.75C_2_H_3_N, and ({[(2,2-bis­{[(2-oxido­benzyl­idene)amino-κ^2^ *N*,*O*]meth­yl}prop­yl)imino]­meth­yl}phenololato-κ^2^ *N*,*O*)indium(III) di­chloro­methane monosolvate, [In(C_26_H_24_N_3_O_3_)]·CH_2_Cl_2_. All three metal complexes are pseudo-octa­hedral and each structure contains multiple weak C—H⋯O and/or C—H⋯N inter­molecular hydrogen-bonding inter­actions. The syntheses and additional characterization in the forms of melting points, high-resolution mass spectra, infra-red (IR) spectra, and ^1^H and ^13^C NMR spectra are also reported.

## Abstract 2

The crystal structures of gallium and indium coordinated by the sexadentate ligand 1,1,1-tris­[(salicyl­idene­amino)­meth­yl]ethane are presented as different solvates. The syntheses, melting points, infra-red (IR) spectra, high-resolution mass spectra, and ^1^H and ^13^ NMR spectra are also reported.

## Chemical context   

**Figure 1:**
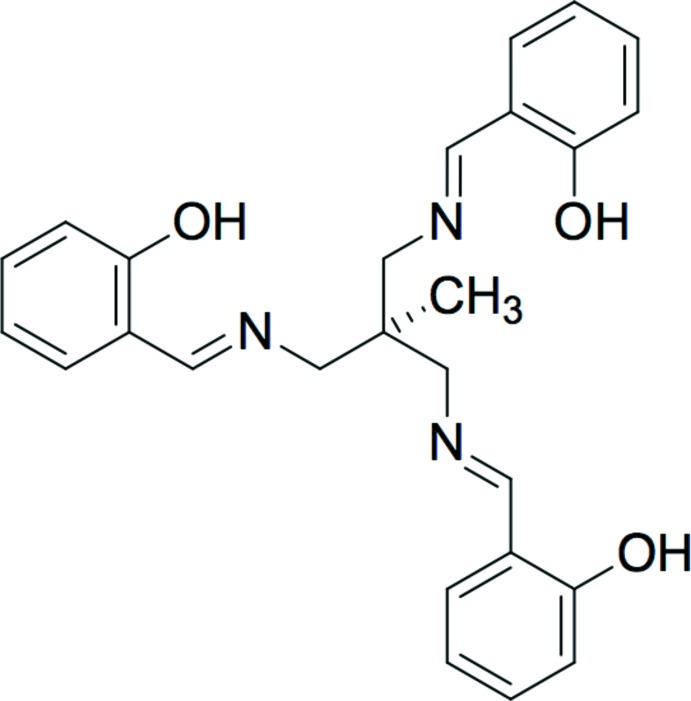
Drawing of 1,1,1-tris­((salicyl­idene­amino)­meth­yl)ethane, H_3_(sal)_3_tame. Deprotonation at the three hydroxyl sites allows for a trianionic, sexadentate ligand.

The synthesis of the sexadentate ligand, 1,1,1-tris­[(salicyl­idene­amino)­meth­yl]ethane, H_3_(sal)_3_tame (Fig. 1 ▸) was first reported nearly fifty years ago (Johnston, 1974 ▸), although its structure was published recently (Yamaguchi *et al.*, 2008*b* ▸). Complexes of the triply deprotonated ligand, (sal)_3_tame, have been reported with transition metals and lanthanides (Sunatsuki *et al.*, 2008 ▸; Yamaguchi *et al.*, 2004 ▸, 2008*a* ▸,*b* ▸; Yokoyama *et al.*, 2010 ▸; Kojima, 2000 ▸; Kobayashi *et al.*, 2006 ▸; Urushigawa *et al.*, 1977 ▸), but have received little attention to date with main-group elements (Katsuta *et al.*, 2012 ▸; Kojima *et al.*, 2000 ▸). The H_3_(sal)_3_tame ligand has already been used to synthesize potential technetium radiopharmaceuticals (Marmion *et al.*, 1996 ▸). There has also been inter­est in polydentate ligands in indium and gallium complexes to be used in radiopharmaceuticals, positron emission tomography, and fluorescence imaging (Liu *et al.*, 1993*a* ▸,*b* ▸; Green *et al.*, 1984 ▸; Liu *et al.*, 1992 ▸; Moerlein & Welch, 1981 ▸; Evans & Jakubovic, 1988 ▸; Zhang *et al.*, 1992 ▸; Gut & Holland, 2019 ▸; Arrowsmith *et al.*, 2011 ▸). Herein we report of the syntheses of the title compounds in good yields along with their respective crystal structures.
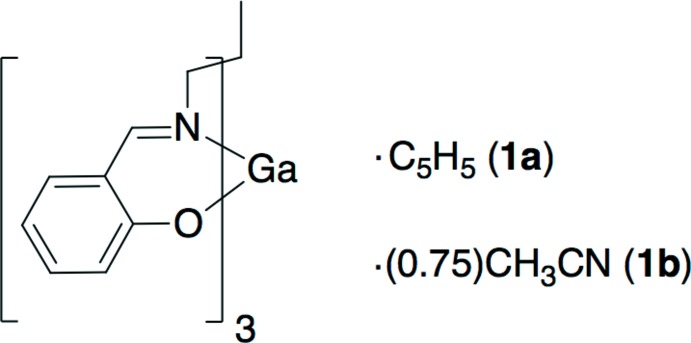
 
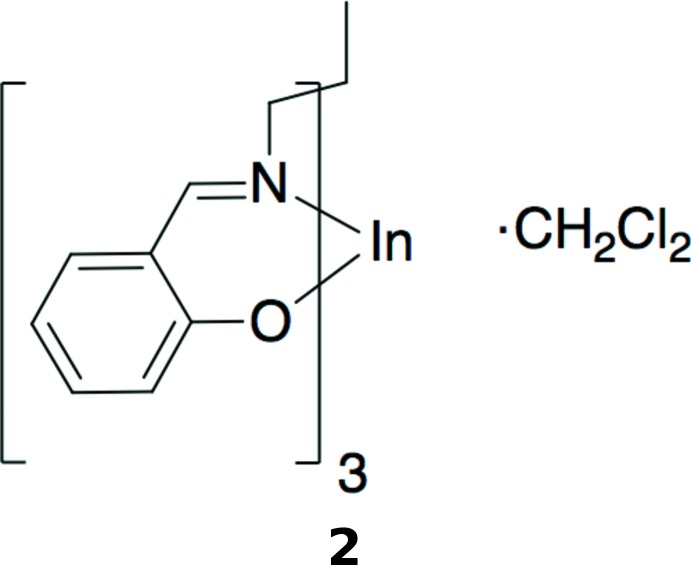


## Structural commentary   

**Figure 2:**
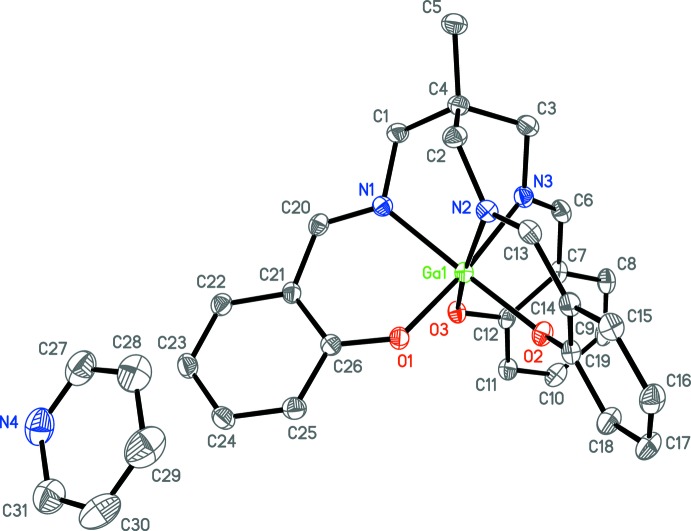
Anisotropic displacement ellipsoid plot of **1a** drawn at the 50% probability level with hydrogen atoms omitted.

**Figure 3:**
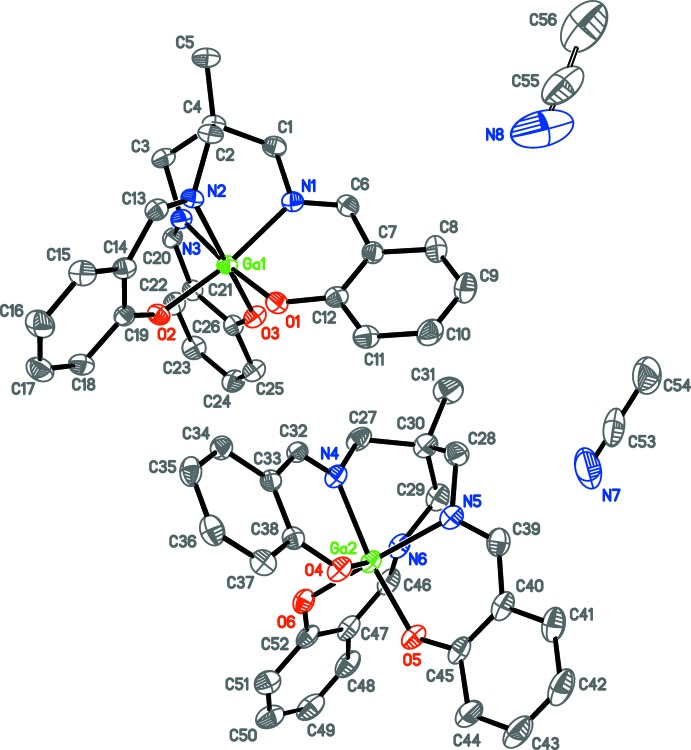
Anisotropic displacement ellipsoid plot of **1b** drawn at the 50% probability level with hydrogen atoms omitted. Only one position of the solvent mol­ecule N8–C55–C56 is shown. The other position is generated by the inversion-symmetry operation −*x*, 1 − *y*, 1 − *z*.

**Figure 4:**
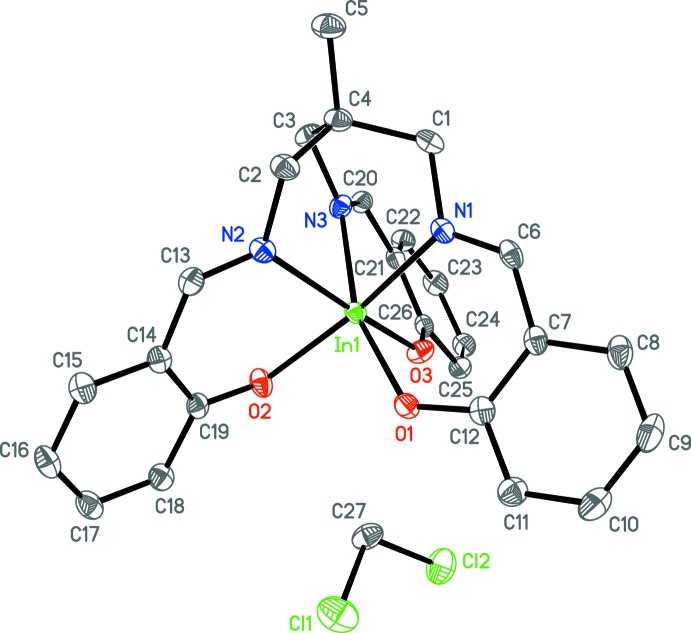
Anisotropic displacement ellipsoid plot of **2** drawn at the 50% probability level with hydrogen atoms omitted.

The asymmetric unit of **1a** (Fig. 2 ▸) contains the gallium center, the (sal)_3_tame ligand, and one co-crystallized pyridine solvent mol­ecule, all in general positions. The geometry is pseudo-octa­hedral, with the smaller angles ranging from 82.13 (6) to 95.97 (6)° (Table 1 ▸). The average Ga—N and Ga–O bond lengths are 2.071 (3) and 1.924 (2) Å, respectively, similar to those found in the the structure of the analogous Ga mol­ecule with a (sal)_3_tame-O-*iso*-Bu ligand [2.080 (5) and 1.916 (3) Å; Green *et al.*, 1993 ▸]. The asymmetric unit of **1b** (Fig. 3 ▸) contains two independent [(sal)_3_tame]gallium complexes and one co-crystallized aceto­nitrile solvent mol­ecule in general positions and one-half of a co-crystallized acetonitile solvent mol­ecule on a crystallographic inversion center. Analogous bond lengths and angles of the two metal complexes of **1b** are nearly identical with each other (Table 2 ▸) and to those of **1a**. The geometry is also pseudo-octa­hedral with the smaller angles ranging from 82.74 (6) to 95.36 (6)° and 82.12 (7) to 97.10 (6)° for the two mol­ecules. The indium analog **2** (Fig. 4 ▸) has the metal center, one (sal)_3_tame ligand, and one co-crystallized di­chloro­methane solvent mol­ecule in general positions in its asymmetric unit. The geometry is more distorted from octa­hedral (Table 3 ▸) than found in mol­ecules **1a** and **1b** with angles ranging from 82.19 (5) to 105.02 (5)°, but consistent with those found in known mol­ecules of indium with (sal)_3_tame ligands that are substituted at the second ethane carbon atom (Gottschaldt *et al.*, 2009 ▸), likely due to the larger effective ionic radius of six-coordinate indium(III) (0.94 Å) *versus* gallium(III) (0.76 Å; Shannon, 1976 ▸).

## Supra­molecular features   

**Figure 5:**
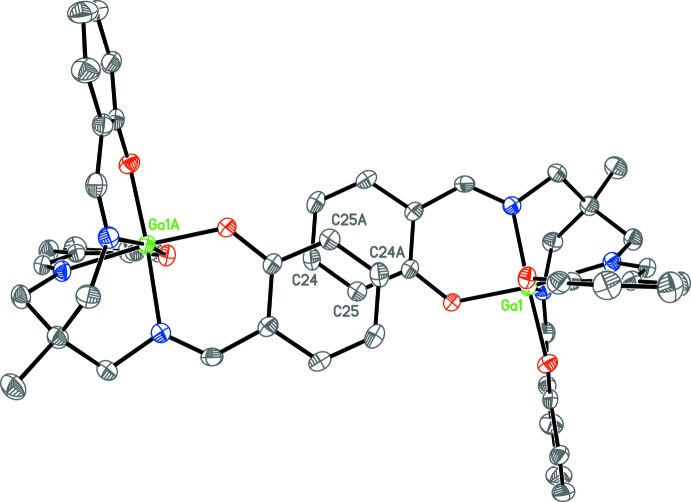
Anisotropic displacement ellipsoid plot of one Ga mol­ecule of **1b** and its inversion-symmetry equivalent (1 − *x*, −*y*, −*z*) drawn at the 50% probability level. Only one edge of the featured rings are overlapped, with a plane separation of approximately 3.3 Å.

All three structures have multiple weak C—H⋯O and/or C—H⋯N hydrogen bonds. These are listed in Tables 4 ▸–6 ▸ ▸, respectively, for the three structures. The ring systems were also examined for possible π–π inter­actions. In **1a**, the phenyl ring C21–C26 is adjacent to the pyridine solvent mol­ecule, with atom C24 being at a distance of 3.429 (3) Å from the pyridine ring plane; however, the angle between their planes of 26.09 (9)° directs the π orbitals away from the other ring. There is a partial overlap of parallel rings in **1b**. Atoms C24 and C25 overlap their inversion-symmetry equivalents (1 − *x*, −*y*, −*z*) at a plane–plane distance of approximately 3.3 Å (Fig. 5 ▸).

## Database survey   

There are two instances of the unsubstituted (sal)_3_tame ligand coordinated to a single metal center in a sexadentate manner found in the Cambridge Structural Database (CSD, Version 5.41, November 2019 update; Groom *et al.*, 2016 ▸). One is a manganese cation (refcode YUKCOW; Drew *et al.*, 1995 ▸) and the other is a neutral iron complex (refcode NOZJER; Deeney *et al.*, 1998 ▸). If substitution is allowed at the second carbon of the ethane moiety, there are six additional structures, two of which contain the main-group elements Ga and In as mentioned above (see *Structural commentary*). If substitution is allowed on the phenyl rings, ten additional structures are found, including one with Ga (refcode CIWXIP; Green *et al.*, 1984 ▸). With bridging allowed at the oxygen sites, 24 additional multimetallic structures are found, but none are with main-group metals.

## Synthesis and crystallization   

The H_3_(sal)_3_tame ligand was synthesized *via* literature procedures [Liu *et al.*, 1993*a* ▸; Kojima *et al.*, 2000 ▸; Robards & Patsalides, 1999 ▸; Marmion *et al.*, 1996 ▸ (^1^H NMR spectra); Ohta *et al.*, 2001 ▸].

[(Sal)_3_tame]gallium(III), **1**. 0.050 g of H_3_(sal)_3_tame ligand (0.12 mmol) were stirred in 10 mL of methanol under an N_2_(g) atmosphere. 0.030 g of gallium(III) nitrate hydrate (0.12 mmol) in 10 mL of degassed methanol was added dropwise to the ligand solution along with 0.5 mL of tri­ethyl­amine. This was stirred at room temperature under N_2_ for 45 minutes. The white solid was filtered and washed with water and methanol. Yield: 0.034 g (61%). M.p. 613–618 K (dec.). IR (neat), ν (cm^−1^): 2907, 1643, 1621, 1598, 1536, 1468, 1445, 1394, 1336, 1308, 1198, 1146, 1024, 893, 761. ^1^H NMR (400 MHz, DMSO-*d*_6_, δ, ppm): 1.09 (*s*, 3H), 3.46 (*d*, 3H, *J* = 14.0 Hz), 4.06 (*d*, 3H, *J* = 13.6 Hz), 6.47 (*d*, 3H, *J* = 8.0 Hz), 6.55 (*t*, 3H, *J* = 7.6 Hz), 7.15–7.23 (*m*, 6H), 8.29 (*s*, 3H). ^13^C NMR (100 MHz, DMSO-*d*_6_, δ, ppm): 23.1, 34.9, 65.8, 114.6, 119.2, 122.3, 134.4, 134.6, 168.7, 169.9. Calculated for C_26_H_24_N_3_O_3_GaNa: 518.10. Found: 518.10. The solid material was dissolved in pyridine (**1a**) or aceto­nitrile (**1b**), and hexa­nes were diffused into the solution to give light-yellow single crystalline blocks.

[(Sal)_3_tame]indium(III), **2**. 0.037 g of H_3_(sal)_3_tame ligand (0.09 mmol) were stirred in 10 mL of methanol under an N_2_(g) atmosphere. 0.019 g of indium chloride (0.09 mmol) in 10 mL of degassed methanol was added dropwise to the ligand solution along with 0.5 mL of tri­ethyl­amine. This was stirred at room temperature under N_2_ for 45 minutes and allowed to sit overnight. The light-yellow solid was filtered and washed with water and methanol. Yield: 0.0322 g (69%). M.p. 658–663 K. IR (neat), ν (cm^−1^): 2914, 1617, 1537, 1465, 1441, 1398, 1347, 1306, 1191, 1019, 893, 761. ^1^H NMR (400 MHz, DMSO-*d*_6_, δ, ppm): 1.09 (*s*, 3H), 3.83 (*s*, 6H), 6.56 (*t*, 3H, *J* = 8.0 Hz), 6.62 (*d*, 3H, *J* = 10.5 Hz), 7.19–7.23 (*m*, 6H), 8.37 (*s*, 3H). ^13^C NMR (100 MHz, DMSO-*d*_6_, δ, ppm): 24.5, 36.6, 67.4, 114.8, 119.1, 123.1, 134.5, 135.9, 170.6, 173.3. Calculated for C_26_H_24_N_3_O_3_InNa: 564.07. Found: 564.08. The solid material was dissolved in di­chloro­methane, and hexa­nes were diffused into the solution to give colorless single crystalline blocks.

## Refinement   

Crystal data, data collection and structure refinement details are summarized in Table 7 ▸. Aceto­nitrile mol­ecule N8–C55–C56 in **1b** was modeled as disordered over a crystallographic inversion center (0.50:0.50). Analogous bond lengths of the disordered solvent mol­ecule were restrained to be similar to those of the ordered solvent mol­ecule (N7–C53–C54). Anisotropic displacement parameters were heavily restrained toward the expected, realistic thermal motion of each atom along the solvent mol­ecule (*SHELXL* hard restraint ‘RIGU’; Thorn *et al.*, 2012 ▸).

All H atoms were refined using riding models. In **1a** and **1b**: aromatic and *sp* ^2^ C—H = 0.95 Å, methyl­ene C—H = 0.99 Å, with *U*_iso_(H) = 1.2*U*_eq_(C), and methyl C—H = 0.98 Å, with *U*_iso_(H) = 1.5*U*_eq_(C). In **2**: aromatic and *sp* ^2^ C—H = 0.93 Å, methyl­ene C—H = 0.97 Å, with *U*_iso_(H) = 1.2*U*_eq_(C), and methyl C—H = 0.96 Å, with *U*_iso_(H) = 1.5*U*_eq_(C).

In **1a** the maximum residual peak of 0.63 e^−^ Å^−3^ and the deepest hole of −0.59 e^−^ Å^−3^ are found 0.94 and 0.65 Å from atoms O2 and Ga1, respectively.

In **1b** the maximum residual peak of 0.62 e^−^ Å^−3^ and the deepest hole of −0.55 e^−^ Å^−3^ are found 0.83 and 0.58 Å from atoms C54 and Ga2, respectively.

In **2** the maximum residual peak of 0.62 e^−^ Å^−3^ and the deepest hole of −0.53 e^−^ Å^−3^ are found 0.73 and 0.56 Å, respectively, from atom Cl2.

## Supplementary Material

Crystal structure: contains datablock(s) 1a, 1b, 2, global. DOI: 10.1107/S2056989020004375/ex2030sup1.cif

Structure factors: contains datablock(s) 1a. DOI: 10.1107/S2056989020004375/ex20301asup2.hkl

Structure factors: contains datablock(s) 1b. DOI: 10.1107/S2056989020004375/ex20301bsup3.hkl

Structure factors: contains datablock(s) 2. DOI: 10.1107/S2056989020004375/ex20302sup4.hkl

CCDC references: 1993782, 1993781, 1993780

Additional supporting information:  crystallographic information; 3D view; checkCIF report

## Figures and Tables

**Table 1:** Selected geometric parameters (Å, °) for **1a** [Chem scheme1]

Ga1—O2	1.9177 (13)	Ga1—N1	2.0500 (16)
Ga1—O1	1.9201 (13)	Ga1—N2	2.0700 (16)
Ga1—O3	1.9331 (13)	Ga1—N3	2.0923 (16)
			
O2—Ga1—O1	90.08 (6)	O3—Ga1—N2	168.56 (6)
O2—Ga1—O3	90.59 (6)	N1—Ga1—N2	86.39 (6)
O1—Ga1—O3	95.47 (6)	O2—Ga1—N3	95.21 (6)
O2—Ga1—N1	175.18 (6)	O1—Ga1—N3	174.34 (6)
O1—Ga1—N1	88.81 (6)	O3—Ga1—N3	86.51 (6)
O3—Ga1—N1	94.19 (6)	N1—Ga1—N3	85.75 (6)
O2—Ga1—N2	89.06 (6)	N2—Ga1—N3	82.13 (6)
O1—Ga1—N2	95.97 (6)		

**Table 2:** Selected geometric parameters (Å, °) for **1b** [Chem scheme1]

Ga1—O3	1.9175 (13)	Ga2—O6	1.9238 (14)
Ga1—O1	1.9215 (13)	Ga2—O5	1.9239 (14)
Ga1—O2	1.9302 (13)	Ga2—O4	1.9296 (13)
Ga1—N1	2.0668 (16)	Ga2—N4	2.0583 (16)
Ga1—N3	2.0719 (16)	Ga2—N5	2.0897 (18)
Ga1—N2	2.0976 (16)	Ga2—N6	2.0984 (16)
			
O3—Ga1—O1	92.70 (6)	O6—Ga2—O5	91.00 (6)
O3—Ga1—O2	94.28 (6)	O6—Ga2—O4	93.51 (6)
O1—Ga1—O2	91.39 (6)	O5—Ga2—O4	91.22 (6)
O3—Ga1—N1	95.36 (6)	O6—Ga2—N4	96.64 (6)
O1—Ga1—N1	89.77 (6)	O5—Ga2—N4	172.32 (7)
O2—Ga1—N1	170.22 (6)	O4—Ga2—N4	89.15 (6)
O3—Ga1—N3	89.22 (6)	O6—Ga2—N5	169.36 (6)
O1—Ga1—N3	174.65 (6)	O5—Ga2—N5	87.99 (6)
O2—Ga1—N3	93.44 (6)	O4—Ga2—N5	97.10 (6)
N1—Ga1—N3	85.08 (6)	N4—Ga2—N5	84.35 (7)
O3—Ga1—N2	174.19 (6)	O6—Ga2—N6	87.38 (6)
O1—Ga1—N2	92.79 (6)	O5—Ga2—N6	93.86 (6)
O2—Ga1—N2	87.50 (6)	O4—Ga2—N6	174.83 (6)
N1—Ga1—N2	82.74 (6)	N4—Ga2—N6	85.69 (6)
N3—Ga1—N2	85.15 (6)	N5—Ga2—N6	82.12 (7)

**Table 3:** Selected geometric parameters (Å, °) for **2** [Chem scheme1]

In1—O1	2.1027 (11)	In1—N1	2.2365 (14)
In1—O2	2.0935 (11)	In1—N2	2.2458 (13)
In1—O3	2.1020 (11)	In1—N3	2.2453 (13)
			
O1—In1—N1	84.46 (5)	O3—In1—O1	89.18 (4)
O1—In1—N2	105.02 (5)	O3—In1—N1	102.84 (5)
O1—In1—N3	162.70 (5)	O3—In1—N2	165.28 (5)
O2—In1—O1	92.35 (5)	O3—In1—N3	84.66 (5)
O2—In1—O3	91.97 (5)	N1—In1—N2	82.75 (5)
O2—In1—N1	164.77 (5)	N1—In1—N3	81.19 (5)
O2—In1—N2	83.71 (5)	N3—In1—N2	82.75 (5)
O2—In1—N3	103.97 (5)		

**Table 4:** Hydrogen-bond geometry (Å, °) for **1a** [Chem scheme1]

*D*—H⋯*A*	*D*—H	H⋯*A*	*D*⋯*A*	*D*—H⋯*A*
C15—H15⋯O1^i^	0.95	2.96	3.540 (2)	121
C16—H16⋯O1^i^	0.95	2.83	3.462 (2)	125
C20—H20⋯O1^ii^	0.95	2.78	3.552 (2)	139
C22—H22⋯O1^ii^	0.95	2.70	3.391 (2)	130
C20—H20⋯O3^ii^	0.95	2.36	3.233 (2)	153
C8—H8⋯O2^iii^	0.95	2.58	3.502 (2)	164
C22—H22⋯O2^ii^	0.95	2.87	3.812 (2)	172

Symmetry codes: (i) 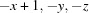; (ii) 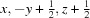; (iii) 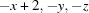.

**Table 5:** Hydrogen-bond geometry (Å, °) for **1b** [Chem scheme1]

*D*—H⋯*A*	*D*—H	H⋯*A*	*D*⋯*A*	*D*—H⋯*A*
C32—H32⋯O1	0.95	2.51	3.333 (2)	146
C34—H34⋯O1	0.95	2.88	3.574 (2)	131
C15—H15⋯O1^i^	0.95	2.65	3.499 (2)	149
C24—H24⋯O2^ii^	0.95	2.83	3.610 (2)	140
C54—H54*B*⋯O2^iii^	0.98	2.31	3.282 (3)	171
C27—H27*A*⋯O3	0.99	2.89	3.697 (2)	140
C6—H6⋯O4^iv^	0.95	2.68	3.557 (2)	153
C8—H8⋯O4^iv^	0.95	2.84	3.642 (3)	143
C8—H8⋯O5^iv^	0.95	2.91	3.806 (3)	157
C48—H48⋯O5^v^	0.95	2.55	3.413 (3)	151
C6—H6⋯O6^iv^	0.95	2.54	3.325 (2)	140
C22—H22⋯O6^ii^	0.95	2.56	3.502 (2)	173
C28—H28*A*⋯N7	0.99	2.91	3.680 (4)	135
C29—H29*B*⋯N7	0.99	2.72	3.554 (4)	143
C31—H31*C*⋯N7	0.98	2.85	3.703 (4)	146
C10—H10⋯N8^vi^	0.95	2.63	3.508 (7)	154

Symmetry codes: (i) 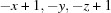; (ii) 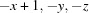; (iii) 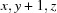; (iv) 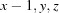; (v) 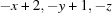; (vi) 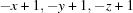.

**Table 6:** Hydrogen-bond geometry (Å, °) for **2** [Chem scheme1]

*D*—H⋯*A*	*D*—H	H⋯*A*	*D*⋯*A*	*D*—H⋯*A*
C6—H6⋯O2^i^	0.93	2.65	3.3596 (19)	134
C8—H8⋯O2^i^	0.93	2.63	3.394 (2)	139
C27—H27*B*⋯O1	0.97	2.26	3.193 (2)	160
C27—H27*B*⋯O2	0.97	2.82	3.253 (2)	108
C27—H27*B*⋯O3	0.97	2.73	3.411 (2)	127

Symmetry code: (i) 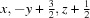.

**Table 7:** Experimental details

	**1a**	**1b**	**2**
Crystal data			
Chemical formula	[Ga(C_26_H_24_N_3_O_3_)]·C_5_H_5_N	[Ga(C_26_H_24_N_3_O_3_)]·0.75C_2_H_3_N	[In(C_26_H_24_N_3_O_3_)]·CH_2_Cl_2_
*M*_r_	575.30	526.99	626.23
Crystal system, space group	Monoclinic, *P*2_1_/*c*	Triclinic, *P* 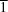	Monoclinic, *P*2_1_/*c*
Temperature (K)	100	173	100
*a*, *b*, *c* (Å)	13.359 (2), 20.413 (3), 9.7470 (15)	10.9053 (6), 14.1157 (8), 16.2324 (9)	10.0704 (2), 16.2514 (4), 16.1749 (4)
α, β, γ (°)	90, 98.326 (3), 90	93.915 (1), 103.120 (1), 97.600 (1)	90, 99.130 (2), 90
*V* (Å^3^)	2629.9 (7)	2399.6 (2)	2613.62 (11)
*Z*	4	4	4
Radiation type	Mo *K*α	Mo *K*α	Mo *K*α
μ (mm^−1^)	1.09	1.18	1.14
Crystal size (mm)	0.24 × 0.12 × 0.10	0.24 × 0.24 × 0.20	0.34 × 0.14 × 0.07

Data collection			
Diffractometer	Bruker SMART APEXII CCD platform	Bruker SMART APEXII CCD platform	XtaLAB Synergy, Dualflex, HyPix
Absorption correction	Multi-scan (*SADABS*; Sheldrick, 1996 ▸)	Multi-scan (*SADABS*; Sheldrick, 1996 ▸)	Multi-scan (*CrysAlis PRO*; Rigaku OD, 2019 ▸)
*T*_min_, *T*_max_	0.645, 0.748	0.666, 0.748	0.676, 1.000
No. of measured, independent and observed [*I* > 2σ(*I*)] reflections	60216, 12727, 7432	52020, 20841, 12632	31229, 8621, 7401
*R*_int_	0.108	0.056	0.037
(sin θ/λ)_max_ (Å^−1^)	0.833	0.806	0.768

Refinement			
*R*[*F* ^2^ > 2σ(*F* ^2^)], *wR*(*F* ^2^), *S*	0.050, 0.120, 1.00	0.048, 0.119, 1.00	0.029, 0.064, 1.06
No. of reflections	12727	20841	8621
No. of parameters	353	653	326
No. of restraints	0	12	0
H-atom treatment	H-atom parameters constrained	H-atom parameters constrained	H-atom parameters constrained
Δρ_max_, Δρ_min_ (e Å^−3^)	0.63, −0.60	0.62, −0.55	0.62, −0.53

Computer programs: *APEX2* (Bruker, 2011 ▸), *SAINT* (Bruker, 2009 ▸), *CrysAlis PRO* (Rigaku OD, 2019 ▸), *SIR97* (Altomare *et al.*, 1999 ▸), *SHELXT* (Sheldrick, 2015*a* ▸), *SHELXL* (Sheldrick, 2015*b* ▸), and *OLEX2* (Dolomanov *et al.*, 2009 ▸).

## supplementary crystallographic information

### ({[(2,2-Bis{[(2-oxidobenzylidene)amino-κ2N,O]methyl}propyl)imino]methyl}phenololato-κ2N,O)gallium(III) pyridine monosolvate (1a) Crystal data

**Table:**

[Ga(C_26_H_24_N_3_O_3_)]·C_5_H_5_N	*F*(000) = 1192
*M**_r_* = 575.30	*D*_x_ = 1.453 Mg m^−^^3^
Monoclinic, *P*2_1_/*c*	Mo *K*α radiation, λ = 0.71073 Å
*a* = 13.359 (2) Å	Cell parameters from 4038 reflections
*b* = 20.413 (3) Å	θ = 2.5–29.3°
*c* = 9.7470 (15) Å	µ = 1.09 mm^−^^1^
β = 98.326 (3)°	*T* = 100 K
*V* = 2629.9 (7) Å^3^	Block, light yellow-red
*Z* = 4	0.24 × 0.12 × 0.10 mm

### ({[(2,2-Bis{[(2-oxidobenzylidene)amino-κ2N,O]methyl}propyl)imino]methyl}phenololato-κ2N,O)gallium(III) pyridine monosolvate (1a) Data collection

**Table:**

Bruker SMART APEXII CCD platform diffractometer	12727 independent reflections
Radiation source: fine-focus sealed tube	7432 reflections with *I* > 2σ(*I*)
Graphite monochromator	*R*_int_ = 0.108
ω scans	θ_max_ = 36.3°, θ_min_ = 1.8°
Absorption correction: multi-scan (SADABS; Sheldrick, 1996)	*h* = −22→22
*T*_min_ = 0.645, *T*_max_ = 0.748	*k* = −34→33
60216 measured reflections	*l* = −16→16

### ({[(2,2-Bis{[(2-oxidobenzylidene)amino-κ2N,O]methyl}propyl)imino]methyl}phenololato-κ2N,O)gallium(III) pyridine monosolvate (1a) Refinement

**Table:**

Refinement on *F*^2^	Primary atom site location: structure-invariant direct methods
Least-squares matrix: full	Secondary atom site location: difference Fourier map
*R*[*F*^2^ > 2σ(*F*^2^)] = 0.050	Hydrogen site location: inferred from neighbouring sites
*wR*(*F*^2^) = 0.120	H-atom parameters constrained
*S* = 1.00	*w* = 1/[σ^2^(*F*_o_^2^) + (0.0454*P*)^2^] where *P* = (*F*_o_^2^ + 2*F*_c_^2^)/3
12727 reflections	(Δ/σ)_max_ = 0.001
353 parameters	Δρ_max_ = 0.63 e Å^−^^3^
0 restraints	Δρ_min_ = −0.59 e Å^−^^3^

### ({[(2,2-Bis{[(2-oxidobenzylidene)amino-κ2N,O]methyl}propyl)imino]methyl}phenololato-κ2N,O)gallium(III) pyridine monosolvate (1a) Special details

**Table:**

Geometry. All esds (except the esd in the dihedral angle between two l.s. planes) are estimated using the full covariance matrix. The cell esds are taken into account individually in the estimation of esds in distances, angles and torsion angles; correlations between esds in cell parameters are only used when they are defined by crystal symmetry. An approximate (isotropic) treatment of cell esds is used for estimating esds involving l.s. planes.

### ({[(2,2-Bis{[(2-oxidobenzylidene)amino-κ2N,O]methyl}propyl)imino]methyl}phenololato-κ2N,O)gallium(III) pyridine monosolvate (1a) Fractional atomic coordinates and isotropic or equivalent isotropic displacement parameters (Å^2^)

**Table:**

	*x*	*y*	*z*	*U*_iso_*/*U*_eq_	
Ga1	0.77587 (2)	0.11922 (2)	0.10318 (2)	0.01430 (5)	
O1	0.65211 (9)	0.16149 (6)	0.02567 (13)	0.0164 (3)	
O2	0.76170 (10)	0.05883 (6)	−0.04912 (14)	0.0176 (3)	
O3	0.86274 (9)	0.17434 (6)	0.01029 (14)	0.0165 (2)	
N1	0.78187 (11)	0.17932 (7)	0.27268 (16)	0.0156 (3)	
N2	0.70942 (11)	0.05079 (7)	0.21825 (16)	0.0164 (3)	
N3	0.90865 (12)	0.07722 (7)	0.20645 (16)	0.0168 (3)	
C1	0.84162 (14)	0.15801 (9)	0.40364 (19)	0.0179 (3)	
H1A	0.817473	0.180960	0.482120	0.021*	
H1B	0.913552	0.169687	0.403699	0.021*	
C2	0.72347 (14)	0.06027 (10)	0.36926 (19)	0.0186 (4)	
H2A	0.709864	0.018585	0.415092	0.022*	
H2B	0.674714	0.093384	0.393262	0.022*	
C3	0.90770 (14)	0.04766 (9)	0.3439 (2)	0.0193 (4)	
H3A	0.976258	0.050283	0.398017	0.023*	
H3B	0.888897	0.000841	0.332989	0.023*	
C4	0.83209 (14)	0.08310 (9)	0.42247 (19)	0.0172 (3)	
C5	0.85760 (15)	0.06699 (10)	0.5761 (2)	0.0220 (4)	
H5A	0.925490	0.083305	0.611104	0.033*	
H5B	0.855577	0.019421	0.588921	0.033*	
H5C	0.808143	0.087887	0.627054	0.033*	
C6	0.98866 (14)	0.07028 (9)	0.1487 (2)	0.0198 (4)	
H6	1.040997	0.043083	0.193998	0.024*	
C7	1.00473 (14)	0.10078 (9)	0.0203 (2)	0.0180 (4)	
C8	1.08968 (14)	0.08049 (9)	−0.0384 (2)	0.0209 (4)	
H8	1.130945	0.046128	0.004245	0.025*	
C9	1.11419 (15)	0.10946 (10)	−0.1567 (2)	0.0237 (4)	
H9	1.170891	0.094779	−0.196828	0.028*	
C10	1.05410 (15)	0.16086 (10)	−0.2165 (2)	0.0219 (4)	
H10	1.070829	0.181452	−0.297569	0.026*	
C11	0.97104 (14)	0.18234 (9)	−0.1603 (2)	0.0192 (4)	
H11	0.932365	0.217933	−0.202536	0.023*	
C12	0.94230 (13)	0.15249 (9)	−0.04132 (19)	0.0158 (3)	
C13	0.65794 (13)	0.00093 (9)	0.1673 (2)	0.0169 (3)	
H13	0.628966	−0.026250	0.230209	0.020*	
C14	0.64051 (13)	−0.01719 (9)	0.02280 (19)	0.0166 (3)	
C15	0.57195 (14)	−0.06808 (9)	−0.0174 (2)	0.0191 (4)	
H15	0.540261	−0.089511	0.051352	0.023*	
C16	0.54908 (15)	−0.08796 (10)	−0.1531 (2)	0.0219 (4)	
H16	0.502691	−0.122701	−0.178095	0.026*	
C17	0.59570 (16)	−0.05586 (10)	−0.2530 (2)	0.0228 (4)	
H17	0.579623	−0.068209	−0.347598	0.027*	
C18	0.66476 (16)	−0.00649 (10)	−0.2167 (2)	0.0225 (4)	
H18	0.695669	0.014241	−0.287057	0.027*	
C19	0.69088 (14)	0.01416 (9)	−0.0783 (2)	0.0162 (3)	
C20	0.73714 (13)	0.23531 (9)	0.27275 (19)	0.0165 (3)	
H20	0.752561	0.261800	0.353170	0.020*	
C21	0.66561 (13)	0.26093 (9)	0.16062 (19)	0.0156 (3)	
C22	0.62965 (15)	0.32520 (9)	0.1758 (2)	0.0189 (4)	
H22	0.659486	0.351438	0.251318	0.023*	
C23	0.55173 (15)	0.35035 (10)	0.0823 (2)	0.0215 (4)	
H23	0.529102	0.394032	0.091608	0.026*	
C24	0.50667 (15)	0.31089 (10)	−0.0259 (2)	0.0215 (4)	
H24	0.451558	0.327541	−0.088746	0.026*	
C25	0.54059 (14)	0.24816 (10)	−0.0433 (2)	0.0195 (4)	
H25	0.508355	0.222334	−0.117986	0.023*	
C26	0.62226 (13)	0.22143 (9)	0.04754 (19)	0.0154 (3)	
N4	0.27776 (15)	0.38182 (10)	0.0834 (2)	0.0345 (5)	
C27	0.31372 (18)	0.33322 (12)	0.1663 (3)	0.0339 (5)	
H27	0.356886	0.343928	0.249457	0.041*	
C28	0.29220 (19)	0.26779 (12)	0.1390 (3)	0.0364 (5)	
H28	0.320603	0.234671	0.201213	0.044*	
C31	0.21756 (19)	0.36497 (12)	−0.0326 (3)	0.0350 (5)	
H31	0.191992	0.398827	−0.094817	0.042*	
C30	0.1903 (2)	0.30137 (13)	−0.0673 (3)	0.0411 (6)	
H30	0.145712	0.291911	−0.149997	0.049*	
C29	0.2291 (2)	0.25190 (13)	0.0203 (3)	0.0439 (7)	
H29	0.212361	0.207478	−0.001198	0.053*	

### ({[(2,2-Bis{[(2-oxidobenzylidene)amino-κ2N,O]methyl}propyl)imino]methyl}phenololato-κ2N,O)gallium(III) pyridine monosolvate (1a) Atomic displacement parameters (Å^2^)

**Table:**

	*U*^11^	*U*^22^	*U*^33^	*U*^12^	*U*^13^	*U*^23^
Ga1	0.01435 (9)	0.01324 (9)	0.01559 (9)	0.00030 (8)	0.00307 (6)	−0.00056 (8)
O1	0.0160 (6)	0.0155 (6)	0.0177 (6)	0.0013 (5)	0.0023 (5)	−0.0019 (5)
O2	0.0178 (6)	0.0160 (6)	0.0199 (7)	−0.0024 (5)	0.0053 (5)	−0.0026 (5)
O3	0.0151 (6)	0.0151 (6)	0.0199 (6)	0.0016 (5)	0.0045 (5)	0.0002 (5)
N1	0.0162 (7)	0.0150 (7)	0.0158 (7)	−0.0006 (5)	0.0026 (6)	0.0005 (6)
N2	0.0161 (7)	0.0159 (7)	0.0171 (7)	0.0004 (6)	0.0019 (6)	0.0008 (6)
N3	0.0177 (7)	0.0143 (7)	0.0183 (8)	0.0005 (6)	0.0020 (6)	−0.0001 (6)
C1	0.0199 (8)	0.0174 (8)	0.0152 (8)	−0.0003 (7)	−0.0010 (7)	−0.0008 (7)
C2	0.0207 (9)	0.0196 (8)	0.0160 (9)	−0.0012 (7)	0.0039 (7)	0.0010 (7)
C3	0.0197 (9)	0.0191 (9)	0.0187 (9)	0.0026 (7)	0.0014 (7)	0.0027 (7)
C4	0.0197 (8)	0.0162 (8)	0.0153 (8)	0.0000 (7)	0.0008 (7)	0.0013 (7)
C5	0.0248 (10)	0.0222 (9)	0.0177 (9)	0.0000 (8)	−0.0010 (7)	0.0022 (7)
C6	0.0167 (8)	0.0176 (8)	0.0244 (10)	0.0034 (7)	0.0011 (7)	0.0008 (7)
C7	0.0175 (8)	0.0153 (8)	0.0220 (9)	0.0003 (6)	0.0056 (7)	−0.0028 (7)
C8	0.0173 (8)	0.0176 (8)	0.0285 (10)	0.0018 (7)	0.0061 (7)	−0.0032 (8)
C9	0.0191 (9)	0.0232 (10)	0.0308 (11)	−0.0009 (7)	0.0102 (8)	−0.0084 (8)
C10	0.0222 (9)	0.0231 (9)	0.0219 (10)	−0.0062 (8)	0.0076 (7)	−0.0050 (8)
C11	0.0203 (9)	0.0165 (8)	0.0210 (9)	−0.0002 (7)	0.0041 (7)	−0.0012 (7)
C12	0.0153 (8)	0.0134 (7)	0.0191 (9)	−0.0016 (6)	0.0034 (6)	−0.0039 (6)
C13	0.0146 (8)	0.0158 (8)	0.0200 (9)	0.0003 (6)	0.0022 (7)	0.0026 (7)
C14	0.0155 (8)	0.0147 (8)	0.0194 (9)	0.0006 (6)	0.0017 (7)	−0.0007 (7)
C15	0.0170 (8)	0.0182 (8)	0.0222 (9)	−0.0007 (7)	0.0030 (7)	0.0006 (7)
C16	0.0209 (9)	0.0178 (9)	0.0264 (10)	−0.0040 (7)	0.0017 (8)	−0.0046 (8)
C17	0.0277 (10)	0.0210 (9)	0.0198 (9)	−0.0038 (8)	0.0041 (8)	−0.0068 (7)
C18	0.0289 (10)	0.0196 (9)	0.0199 (9)	−0.0033 (8)	0.0071 (8)	−0.0037 (7)
C19	0.0156 (8)	0.0132 (7)	0.0201 (9)	0.0013 (6)	0.0037 (7)	−0.0009 (6)
C20	0.0176 (8)	0.0159 (8)	0.0171 (8)	−0.0019 (6)	0.0058 (7)	−0.0017 (7)
C21	0.0165 (8)	0.0142 (7)	0.0168 (8)	0.0013 (6)	0.0049 (6)	0.0003 (6)
C22	0.0224 (9)	0.0172 (8)	0.0178 (9)	0.0003 (7)	0.0055 (7)	0.0001 (7)
C23	0.0257 (10)	0.0169 (8)	0.0229 (10)	0.0052 (7)	0.0072 (8)	0.0025 (7)
C24	0.0223 (9)	0.0226 (9)	0.0198 (9)	0.0063 (7)	0.0039 (7)	0.0051 (7)
C25	0.0188 (8)	0.0221 (9)	0.0176 (9)	0.0004 (7)	0.0026 (7)	0.0011 (7)
C26	0.0147 (7)	0.0168 (8)	0.0156 (8)	0.0008 (6)	0.0052 (6)	0.0018 (6)
N4	0.0336 (10)	0.0283 (9)	0.0424 (12)	−0.0019 (9)	0.0080 (9)	−0.0097 (9)
C27	0.0279 (11)	0.0397 (13)	0.0345 (13)	−0.0025 (10)	0.0057 (10)	−0.0124 (11)
C28	0.0398 (14)	0.0325 (12)	0.0385 (14)	−0.0027 (10)	0.0113 (11)	−0.0008 (10)
C31	0.0390 (13)	0.0315 (12)	0.0352 (13)	−0.0004 (10)	0.0074 (10)	−0.0033 (10)
C30	0.0490 (15)	0.0442 (15)	0.0303 (13)	−0.0139 (13)	0.0061 (11)	−0.0122 (11)
C29	0.0610 (18)	0.0294 (13)	0.0419 (15)	−0.0146 (12)	0.0100 (13)	−0.0088 (11)

### ({[(2,2-Bis{[(2-oxidobenzylidene)amino-κ2N,O]methyl}propyl)imino]methyl}phenololato-κ2N,O)gallium(III) pyridine monosolvate (1a) Geometric parameters (Å, º)

**Table:**

Ga1—O2	1.9177 (13)	C11—C12	1.412 (3)
Ga1—O1	1.9201 (13)	C11—H11	0.9500
Ga1—O3	1.9331 (13)	C13—C14	1.442 (3)
Ga1—N1	2.0500 (16)	C13—H13	0.9500
Ga1—N2	2.0700 (16)	C14—C15	1.403 (3)
Ga1—N3	2.0923 (16)	C14—C19	1.423 (3)
O1—C26	1.314 (2)	C15—C16	1.375 (3)
O2—C19	1.315 (2)	C15—H15	0.9500
O3—C12	1.318 (2)	C16—C17	1.393 (3)
N1—C20	1.290 (2)	C16—H16	0.9500
N1—C1	1.470 (2)	C17—C18	1.378 (3)
N2—C13	1.287 (2)	C17—H17	0.9500
N2—C2	1.469 (2)	C18—C19	1.408 (3)
N3—C6	1.286 (2)	C18—H18	0.9500
N3—C3	1.471 (2)	C20—C21	1.442 (3)
C1—C4	1.547 (3)	C20—H20	0.9500
C1—H1A	0.9900	C21—C22	1.412 (3)
C1—H1B	0.9900	C21—C26	1.420 (3)
C2—C4	1.541 (3)	C22—C23	1.379 (3)
C2—H2A	0.9900	C22—H22	0.9500
C2—H2B	0.9900	C23—C24	1.393 (3)
C3—C4	1.534 (3)	C23—H23	0.9500
C3—H3A	0.9900	C24—C25	1.377 (3)
C3—H3B	0.9900	C24—H24	0.9500
C4—C5	1.523 (3)	C25—C26	1.411 (3)
C5—H5A	0.9800	C25—H25	0.9500
C5—H5B	0.9800	N4—C27	1.325 (3)
C5—H5C	0.9800	N4—C31	1.334 (3)
C6—C7	1.442 (3)	C27—C28	1.384 (3)
C6—H6	0.9500	C27—H27	0.9500
C7—C8	1.406 (3)	C28—C29	1.368 (4)
C7—C12	1.423 (3)	C28—H28	0.9500
C8—C9	1.376 (3)	C31—C30	1.377 (3)
C8—H8	0.9500	C31—H31	0.9500
C9—C10	1.396 (3)	C30—C29	1.374 (4)
C9—H9	0.9500	C30—H30	0.9500
C10—C11	1.378 (3)	C29—H29	0.9500
C10—H10	0.9500		
			
O2—Ga1—O1	90.08 (6)	C10—C9—H9	120.6
O2—Ga1—O3	90.59 (6)	C11—C10—C9	121.38 (19)
O1—Ga1—O3	95.47 (6)	C11—C10—H10	119.3
O2—Ga1—N1	175.18 (6)	C9—C10—H10	119.3
O1—Ga1—N1	88.81 (6)	C10—C11—C12	121.29 (18)
O3—Ga1—N1	94.19 (6)	C10—C11—H11	119.4
O2—Ga1—N2	89.06 (6)	C12—C11—H11	119.4
O1—Ga1—N2	95.97 (6)	O3—C12—C11	119.86 (17)
O3—Ga1—N2	168.56 (6)	O3—C12—C7	123.07 (17)
N1—Ga1—N2	86.39 (6)	C11—C12—C7	117.03 (17)
O2—Ga1—N3	95.21 (6)	N2—C13—C14	125.55 (17)
O1—Ga1—N3	174.34 (6)	N2—C13—H13	117.2
O3—Ga1—N3	86.51 (6)	C14—C13—H13	117.2
N1—Ga1—N3	85.75 (6)	C15—C14—C19	119.62 (17)
N2—Ga1—N3	82.13 (6)	C15—C14—C13	118.01 (17)
C26—O1—Ga1	128.24 (12)	C19—C14—C13	122.37 (16)
C19—O2—Ga1	126.91 (12)	C16—C15—C14	122.20 (18)
C12—O3—Ga1	123.63 (11)	C16—C15—H15	118.9
C20—N1—C1	117.07 (16)	C14—C15—H15	118.9
C20—N1—Ga1	124.52 (13)	C15—C16—C17	118.28 (18)
C1—N1—Ga1	118.41 (12)	C15—C16—H16	120.9
C13—N2—C2	118.31 (16)	C17—C16—H16	120.9
C13—N2—Ga1	124.86 (13)	C18—C17—C16	120.99 (19)
C2—N2—Ga1	116.83 (12)	C18—C17—H17	119.5
C6—N3—C3	118.08 (16)	C16—C17—H17	119.5
C6—N3—Ga1	122.36 (13)	C17—C18—C19	121.96 (19)
C3—N3—Ga1	119.18 (12)	C17—C18—H18	119.0
N1—C1—C4	110.59 (15)	C19—C18—H18	119.0
N1—C1—H1A	109.5	O2—C19—C18	119.07 (17)
C4—C1—H1A	109.5	O2—C19—C14	124.00 (17)
N1—C1—H1B	109.5	C18—C19—C14	116.89 (17)
C4—C1—H1B	109.5	N1—C20—C21	125.27 (17)
H1A—C1—H1B	108.1	N1—C20—H20	117.4
N2—C2—C4	110.76 (15)	C21—C20—H20	117.4
N2—C2—H2A	109.5	C22—C21—C26	120.15 (17)
C4—C2—H2A	109.5	C22—C21—C20	117.11 (17)
N2—C2—H2B	109.5	C26—C21—C20	122.24 (16)
C4—C2—H2B	109.5	C23—C22—C21	120.80 (18)
H2A—C2—H2B	108.1	C23—C22—H22	119.6
N3—C3—C4	110.75 (15)	C21—C22—H22	119.6
N3—C3—H3A	109.5	C22—C23—C24	119.15 (18)
C4—C3—H3A	109.5	C22—C23—H23	120.4
N3—C3—H3B	109.5	C24—C23—H23	120.4
C4—C3—H3B	109.5	C25—C24—C23	121.16 (18)
H3A—C3—H3B	108.1	C25—C24—H24	119.4
C5—C4—C3	108.61 (15)	C23—C24—H24	119.4
C5—C4—C2	109.35 (16)	C24—C25—C26	121.36 (18)
C3—C4—C2	110.20 (15)	C24—C25—H25	119.3
C5—C4—C1	108.63 (15)	C26—C25—H25	119.3
C3—C4—C1	109.59 (15)	O1—C26—C25	118.91 (17)
C2—C4—C1	110.41 (15)	O1—C26—C21	123.76 (16)
C4—C5—H5A	109.5	C25—C26—C21	117.31 (17)
C4—C5—H5B	109.5	C27—N4—C31	116.4 (2)
H5A—C5—H5B	109.5	N4—C27—C28	124.0 (2)
C4—C5—H5C	109.5	N4—C27—H27	118.0
H5A—C5—H5C	109.5	C28—C27—H27	118.0
H5B—C5—H5C	109.5	C29—C28—C27	118.5 (3)
N3—C6—C7	124.76 (17)	C29—C28—H28	120.8
N3—C6—H6	117.6	C27—C28—H28	120.8
C7—C6—H6	117.6	N4—C31—C30	123.8 (3)
C8—C7—C12	120.33 (18)	N4—C31—H31	118.1
C8—C7—C6	117.20 (17)	C30—C31—H31	118.1
C12—C7—C6	122.34 (17)	C29—C30—C31	118.5 (2)
C9—C8—C7	121.23 (19)	C29—C30—H30	120.7
C9—C8—H8	119.4	C31—C30—H30	120.7
C7—C8—H8	119.4	C28—C29—C30	118.8 (2)
C8—C9—C10	118.71 (18)	C28—C29—H29	120.6
C8—C9—H9	120.6	C30—C29—H29	120.6
			
C20—N1—C1—C4	143.42 (16)	C19—C14—C15—C16	−1.8 (3)
Ga1—N1—C1—C4	−37.03 (19)	C13—C14—C15—C16	178.80 (18)
C13—N2—C2—C4	139.46 (17)	C14—C15—C16—C17	−0.3 (3)
Ga1—N2—C2—C4	−40.07 (19)	C15—C16—C17—C18	1.5 (3)
C6—N3—C3—C4	155.81 (17)	C16—C17—C18—C19	−0.5 (3)
Ga1—N3—C3—C4	−31.1 (2)	Ga1—O2—C19—C18	153.46 (14)
N3—C3—C4—C5	−161.58 (15)	Ga1—O2—C19—C14	−29.1 (2)
N3—C3—C4—C2	78.64 (19)	C17—C18—C19—O2	175.99 (18)
N3—C3—C4—C1	−43.0 (2)	C17—C18—C19—C14	−1.6 (3)
N2—C2—C4—C5	−157.84 (15)	C15—C14—C19—O2	−174.77 (17)
N2—C2—C4—C3	−38.5 (2)	C13—C14—C19—O2	4.6 (3)
N2—C2—C4—C1	82.69 (19)	C15—C14—C19—C18	2.7 (3)
N1—C1—C4—C5	−157.85 (15)	C13—C14—C19—C18	−177.91 (17)
N1—C1—C4—C3	83.63 (18)	C1—N1—C20—C21	−171.93 (17)
N1—C1—C4—C2	−37.9 (2)	Ga1—N1—C20—C21	8.6 (3)
C3—N3—C6—C7	−174.51 (18)	N1—C20—C21—C22	−175.78 (17)
Ga1—N3—C6—C7	12.6 (3)	N1—C20—C21—C26	12.3 (3)
N3—C6—C7—C8	−170.91 (19)	C26—C21—C22—C23	0.5 (3)
N3—C6—C7—C12	13.3 (3)	C20—C21—C22—C23	−171.58 (17)
C12—C7—C8—C9	−0.5 (3)	C21—C22—C23—C24	1.7 (3)
C6—C7—C8—C9	−176.32 (19)	C22—C23—C24—C25	−2.0 (3)
C7—C8—C9—C10	1.4 (3)	C23—C24—C25—C26	−0.1 (3)
C8—C9—C10—C11	−0.6 (3)	Ga1—O1—C26—C25	165.16 (13)
C9—C10—C11—C12	−1.1 (3)	Ga1—O1—C26—C21	−16.8 (2)
Ga1—O3—C12—C11	148.16 (14)	C24—C25—C26—O1	−179.51 (17)
Ga1—O3—C12—C7	−34.2 (2)	C24—C25—C26—C21	2.3 (3)
C10—C11—C12—O3	179.70 (17)	C22—C21—C26—O1	179.38 (16)
C10—C11—C12—C7	2.0 (3)	C20—C21—C26—O1	−8.9 (3)
C8—C7—C12—O3	−178.84 (17)	C22—C21—C26—C25	−2.5 (3)
C6—C7—C12—O3	−3.2 (3)	C20—C21—C26—C25	169.20 (17)
C8—C7—C12—C11	−1.2 (3)	C31—N4—C27—C28	−0.1 (4)
C6—C7—C12—C11	174.44 (17)	N4—C27—C28—C29	−0.8 (4)
C2—N2—C13—C14	−177.24 (17)	C27—N4—C31—C30	1.4 (4)
Ga1—N2—C13—C14	2.2 (3)	N4—C31—C30—C29	−1.7 (4)
N2—C13—C14—C15	−171.93 (17)	C27—C28—C29—C30	0.5 (4)
N2—C13—C14—C19	8.7 (3)	C31—C30—C29—C28	0.7 (4)

### ({[(2,2-Bis{[(2-oxidobenzylidene)amino-κ2N,O]methyl}propyl)imino]methyl}phenololato-κ2N,O)gallium(III) pyridine monosolvate (1a) Hydrogen-bond geometry (Å, º)

**Table:**

*D*—H···*A*	*D*—H	H···*A*	*D*···*A*	*D*—H···*A*
C15—H15···O1^i^	0.95	2.96	3.540 (2)	121
C16—H16···O1^i^	0.95	2.83	3.462 (2)	125
C20—H20···O1^ii^	0.95	2.78	3.552 (2)	139
C22—H22···O1^ii^	0.95	2.70	3.391 (2)	130
C20—H20···O3^ii^	0.95	2.36	3.233 (2)	153
C8—H8···O2^iii^	0.95	2.58	3.502 (2)	164
C22—H22···O2^ii^	0.95	2.87	3.812 (2)	172

Symmetry codes: (i) −*x*+1, −*y*, −*z*; (ii) *x*, −*y*+1/2, *z*+1/2; (iii) −*x*+2, −*y*, −*z*.

### ({[(2,2-Bis{[(2-oxidobenzylidene)amino-κ2N,O]methyl}propyl)imino]methyl}phenololato-κ2N,O)gallium(III) acetonitrile 0.75-solvate (1b) Crystal data

**Table:**

[Ga(C_26_H_24_N_3_O_3_)]·0.75C_2_H_3_N	*Z* = 4
*M**_r_* = 526.99	*F*(000) = 1090
Triclinic, *P*1	*D*_x_ = 1.459 Mg m^−^^3^
*a* = 10.9053 (6) Å	Mo *K*α radiation, λ = 0.71073 Å
*b* = 14.1157 (8) Å	Cell parameters from 3787 reflections
*c* = 16.2324 (9) Å	θ = 2.3–31.6°
α = 93.915 (1)°	µ = 1.18 mm^−^^1^
β = 103.120 (1)°	*T* = 173 K
γ = 97.600 (1)°	Block, colorless
*V* = 2399.6 (2) Å^3^	0.24 × 0.24 × 0.20 mm

### ({[(2,2-Bis{[(2-oxidobenzylidene)amino-κ2N,O]methyl}propyl)imino]methyl}phenololato-κ2N,O)gallium(III) acetonitrile 0.75-solvate (1b) Data collection

**Table:**

Bruker SMART APEXII CCD platform diffractometer	20841 independent reflections
Radiation source: fine-focus sealed tube	12632 reflections with *I* > 2σ(*I*)
Graphite monochromator	*R*_int_ = 0.056
ω scans	θ_max_ = 35.0°, θ_min_ = 1.9°
Absorption correction: multi-scan (SADABS; Sheldrick, 1996)	*h* = −17→17
*T*_min_ = 0.666, *T*_max_ = 0.748	*k* = −22→22
52020 measured reflections	*l* = −26→26

### ({[(2,2-Bis{[(2-oxidobenzylidene)amino-κ2N,O]methyl}propyl)imino]methyl}phenololato-κ2N,O)gallium(III) acetonitrile 0.75-solvate (1b) Refinement

**Table:**

Refinement on *F*^2^	Primary atom site location: structure-invariant direct methods
Least-squares matrix: full	Secondary atom site location: difference Fourier map
*R*[*F*^2^ > 2σ(*F*^2^)] = 0.048	Hydrogen site location: inferred from neighbouring sites
*wR*(*F*^2^) = 0.119	H-atom parameters constrained
*S* = 1.00	*w* = 1/[σ^2^(*F*_o_^2^) + (0.0459*P*)^2^] where *P* = (*F*_o_^2^ + 2*F*_c_^2^)/3
20841 reflections	(Δ/σ)_max_ = 0.001
653 parameters	Δρ_max_ = 0.62 e Å^−^^3^
12 restraints	Δρ_min_ = −0.55 e Å^−^^3^

### ({[(2,2-Bis{[(2-oxidobenzylidene)amino-κ2N,O]methyl}propyl)imino]methyl}phenololato-κ2N,O)gallium(III) acetonitrile 0.75-solvate (1b) Special details

**Table:**

Geometry. All esds (except the esd in the dihedral angle between two l.s. planes) are estimated using the full covariance matrix. The cell esds are taken into account individually in the estimation of esds in distances, angles and torsion angles; correlations between esds in cell parameters are only used when they are defined by crystal symmetry. An approximate (isotropic) treatment of cell esds is used for estimating esds involving l.s. planes.
Refinement. One cocrystallized acetonitrile solvent molecule is modeled as disordered over a crystallographic inversion center (50:50). Analogous bond lengths of the disordered solvent molecule were restrained to be similar to those of the ordered solvent molecule. Anisotropic displacement parameters were restrained toward the expected thermal motion of each atom along the solvent molecule.

### ({[(2,2-Bis{[(2-oxidobenzylidene)amino-κ2N,O]methyl}propyl)imino]methyl}phenololato-κ2N,O)gallium(III) acetonitrile 0.75-solvate (1b) Fractional atomic coordinates and isotropic or equivalent isotropic displacement parameters (Å^2^)

**Table:**

	*x*	*y*	*z*	*U*_iso_*/*U*_eq_	Occ. (<1)
Ga1	0.33445 (2)	0.03781 (2)	0.24211 (2)	0.01860 (5)	
O1	0.44828 (12)	0.13910 (10)	0.31777 (8)	0.0220 (3)	
O2	0.46507 (12)	−0.04363 (10)	0.25420 (8)	0.0218 (3)	
O3	0.37262 (13)	0.08983 (10)	0.14315 (8)	0.0241 (3)	
N1	0.18433 (15)	0.11232 (12)	0.24496 (10)	0.0212 (3)	
N2	0.27584 (14)	−0.02693 (12)	0.34216 (10)	0.0209 (3)	
N3	0.19786 (15)	−0.06598 (12)	0.16370 (10)	0.0217 (3)	
C1	0.05468 (18)	0.05719 (15)	0.21985 (13)	0.0255 (4)	
H1A	0.026618	0.046121	0.157090	0.031*	
H1B	−0.005405	0.093924	0.240757	0.031*	
C2	0.14316 (17)	−0.02575 (15)	0.34819 (12)	0.0246 (4)	
H2A	0.135976	0.036435	0.377438	0.030*	
H2B	0.117628	−0.077622	0.381760	0.030*	
C3	0.09945 (18)	−0.11402 (15)	0.20160 (12)	0.0245 (4)	
H3A	0.134205	−0.163818	0.236144	0.029*	
H3B	0.026198	−0.145981	0.155998	0.029*	
C4	0.05462 (18)	−0.04032 (15)	0.25812 (12)	0.0238 (4)	
C5	−0.08040 (19)	−0.08028 (17)	0.26360 (15)	0.0329 (5)	
H5A	−0.082878	−0.146596	0.278286	0.049*	
H5B	−0.139827	−0.079009	0.208585	0.049*	
H5C	−0.104986	−0.040845	0.307452	0.049*	
C6	0.19550 (19)	0.20191 (15)	0.27144 (12)	0.0237 (4)	
H6	0.119766	0.230078	0.263599	0.028*	
C7	0.31410 (19)	0.26305 (14)	0.31215 (12)	0.0232 (4)	
C8	0.3057 (2)	0.35909 (16)	0.33702 (15)	0.0331 (5)	
H8	0.226524	0.381942	0.320075	0.040*	
C9	0.4093 (2)	0.42044 (17)	0.38521 (16)	0.0387 (5)	
H9	0.402551	0.485296	0.400841	0.046*	
C10	0.5245 (2)	0.38591 (16)	0.41078 (14)	0.0336 (5)	
H10	0.596392	0.427516	0.444869	0.040*	
C11	0.5359 (2)	0.29228 (15)	0.38738 (12)	0.0266 (4)	
H11	0.615473	0.270528	0.405827	0.032*	
C12	0.43154 (18)	0.22808 (14)	0.33655 (11)	0.0219 (4)	
C13	0.34944 (18)	−0.06416 (14)	0.40040 (11)	0.0218 (4)	
H13	0.320358	−0.077190	0.449969	0.026*	
C14	0.47214 (18)	−0.08761 (14)	0.39642 (12)	0.0219 (4)	
C15	0.53730 (19)	−0.13112 (16)	0.46534 (13)	0.0277 (4)	
H15	0.506073	−0.132892	0.515377	0.033*	
C16	0.6443 (2)	−0.17097 (18)	0.46246 (14)	0.0339 (5)	
H16	0.687720	−0.199144	0.509935	0.041*	
C17	0.6879 (2)	−0.16919 (17)	0.38805 (14)	0.0329 (5)	
H17	0.760288	−0.198519	0.384291	0.039*	
C18	0.62782 (18)	−0.12554 (15)	0.31982 (13)	0.0260 (4)	
H18	0.660432	−0.124775	0.270303	0.031*	
C19	0.51916 (17)	−0.08206 (13)	0.32185 (12)	0.0204 (4)	
C20	0.18695 (18)	−0.08578 (14)	0.08384 (12)	0.0228 (4)	
H20	0.122273	−0.136873	0.055435	0.027*	
C21	0.26351 (18)	−0.03783 (14)	0.03338 (11)	0.0220 (4)	
C22	0.2445 (2)	−0.07568 (15)	−0.05216 (12)	0.0272 (4)	
H22	0.187717	−0.133888	−0.072312	0.033*	
C23	0.3057 (2)	−0.03073 (16)	−0.10647 (13)	0.0296 (4)	
H23	0.293134	−0.057784	−0.163514	0.036*	
C24	0.3869 (2)	0.05560 (16)	−0.07693 (13)	0.0290 (4)	
H24	0.429615	0.087588	−0.114433	0.035*	
C25	0.4062 (2)	0.09529 (15)	0.00563 (12)	0.0271 (4)	
H25	0.459923	0.155256	0.023510	0.033*	
C26	0.34797 (18)	0.04897 (14)	0.06428 (11)	0.0208 (4)	
Ga2	0.88779 (2)	0.37876 (2)	0.20703 (2)	0.02138 (5)	
O4	0.96341 (13)	0.33951 (10)	0.31629 (8)	0.0252 (3)	
O5	1.03345 (14)	0.47499 (10)	0.21460 (9)	0.0274 (3)	
O6	0.95907 (14)	0.28754 (10)	0.14551 (8)	0.0253 (3)	
N4	0.72159 (16)	0.29010 (12)	0.20611 (10)	0.0234 (3)	
N5	0.79794 (16)	0.48650 (12)	0.25177 (10)	0.0257 (3)	
N6	0.79023 (16)	0.41376 (12)	0.08876 (10)	0.0258 (4)	
C27	0.60495 (19)	0.30705 (15)	0.14703 (14)	0.0301 (5)	
H27A	0.529706	0.275237	0.164450	0.036*	
H27B	0.602541	0.278657	0.089103	0.036*	
C28	0.6574 (2)	0.46920 (16)	0.23465 (14)	0.0304 (4)	
H28A	0.625324	0.531380	0.238360	0.037*	
H28B	0.630943	0.430783	0.278180	0.037*	
C29	0.6718 (2)	0.45548 (16)	0.08224 (13)	0.0314 (5)	
H29A	0.617476	0.439976	0.023830	0.038*	
H29B	0.692541	0.526262	0.093844	0.038*	
C30	0.5993 (2)	0.41555 (16)	0.14597 (13)	0.0298 (4)	
C31	0.4592 (2)	0.4286 (2)	0.11911 (17)	0.0435 (6)	
H31A	0.415714	0.408886	0.163195	0.065*	
H31B	0.418523	0.389050	0.065437	0.065*	
H31C	0.453512	0.496329	0.111512	0.065*	
C32	0.70962 (18)	0.22489 (14)	0.25667 (12)	0.0224 (4)	
H32	0.627549	0.188283	0.249186	0.027*	
C33	0.80972 (18)	0.20277 (14)	0.32341 (12)	0.0213 (4)	
C34	0.7804 (2)	0.12148 (15)	0.36508 (13)	0.0261 (4)	
H34	0.698088	0.084187	0.347472	0.031*	
C35	0.8688 (2)	0.09528 (15)	0.43072 (13)	0.0292 (4)	
H35	0.848965	0.039562	0.457714	0.035*	
C36	0.9883 (2)	0.15185 (16)	0.45706 (13)	0.0296 (4)	
H36	1.050090	0.134068	0.502320	0.036*	
C37	1.0184 (2)	0.23275 (15)	0.41893 (12)	0.0266 (4)	
H37	1.099980	0.270522	0.439196	0.032*	
C38	0.93063 (18)	0.26111 (14)	0.35021 (12)	0.0219 (4)	
C39	0.8561 (2)	0.56736 (16)	0.29109 (13)	0.0302 (4)	
H39	0.805555	0.611047	0.308692	0.036*	
C40	0.9918 (2)	0.59753 (15)	0.31083 (13)	0.0298 (5)	
C41	1.0425 (3)	0.67730 (16)	0.37092 (15)	0.0387 (5)	
H41	0.986699	0.709721	0.395752	0.046*	
C42	1.1712 (3)	0.70961 (18)	0.39470 (17)	0.0466 (7)	
H42	1.204642	0.761936	0.437371	0.056*	
C43	1.2517 (3)	0.66453 (17)	0.35532 (17)	0.0455 (7)	
H43	1.340637	0.687230	0.370669	0.055*	
C44	1.2049 (2)	0.58765 (16)	0.29454 (15)	0.0370 (5)	
H44	1.261587	0.559550	0.267123	0.044*	
C45	1.0733 (2)	0.54965 (14)	0.27215 (13)	0.0273 (4)	
C46	0.8377 (2)	0.40946 (15)	0.02332 (12)	0.0290 (4)	
H46	0.798146	0.438958	−0.024532	0.035*	
C47	0.9459 (2)	0.36367 (15)	0.01634 (13)	0.0284 (4)	
C48	0.9925 (2)	0.37474 (16)	−0.05753 (13)	0.0341 (5)	
H48	0.954108	0.413589	−0.098494	0.041*	
C49	1.0913 (2)	0.33087 (17)	−0.07124 (14)	0.0376 (6)	
H49	1.122712	0.340226	−0.120547	0.045*	
C50	1.1456 (2)	0.27204 (17)	−0.01189 (14)	0.0368 (5)	
H50	1.214005	0.240870	−0.021336	0.044*	
C51	1.1014 (2)	0.25852 (17)	0.06031 (13)	0.0318 (5)	
H51	1.139212	0.217512	0.099417	0.038*	
C52	1.00143 (19)	0.30438 (14)	0.07704 (12)	0.0254 (4)	
N7	0.5915 (4)	0.6858 (2)	0.1290 (2)	0.0948 (12)	
C53	0.5005 (4)	0.71106 (19)	0.13652 (19)	0.0567 (8)	
C54	0.3847 (3)	0.7435 (2)	0.1459 (2)	0.0725 (10)	
H54A	0.326486	0.740988	0.089818	0.109*	
H54B	0.403900	0.809746	0.172898	0.109*	
H54C	0.344580	0.701975	0.181432	0.109*	
N8	0.1723 (7)	0.4753 (6)	0.5239 (4)	0.108 (3)	0.5
C55	0.0695 (12)	0.4891 (11)	0.5101 (7)	0.068 (3)	0.5
C56	−0.0654 (12)	0.4986 (14)	0.4977 (10)	0.092 (4)	0.5
H56A	−0.080115	0.559478	0.474522	0.137*	0.5
H56B	−0.089116	0.497416	0.552425	0.137*	0.5
H56C	−0.117265	0.445106	0.457992	0.137*	0.5

### ({[(2,2-Bis{[(2-oxidobenzylidene)amino-κ2N,O]methyl}propyl)imino]methyl}phenololato-κ2N,O)gallium(III) acetonitrile 0.75-solvate (1b) Atomic displacement parameters (Å^2^)

**Table:**

	*U*^11^	*U*^22^	*U*^33^	*U*^12^	*U*^13^	*U*^23^
Ga1	0.01759 (10)	0.02105 (11)	0.01673 (9)	0.00134 (8)	0.00448 (7)	0.00080 (7)
O1	0.0196 (6)	0.0242 (7)	0.0205 (6)	0.0034 (5)	0.0025 (5)	−0.0019 (5)
O2	0.0220 (6)	0.0265 (7)	0.0182 (6)	0.0061 (5)	0.0062 (5)	0.0024 (5)
O3	0.0292 (7)	0.0235 (7)	0.0176 (6)	−0.0033 (6)	0.0064 (5)	−0.0012 (5)
N1	0.0190 (7)	0.0245 (8)	0.0196 (7)	0.0031 (6)	0.0031 (6)	0.0032 (6)
N2	0.0185 (7)	0.0250 (8)	0.0203 (7)	0.0032 (6)	0.0071 (6)	0.0008 (6)
N3	0.0214 (8)	0.0226 (8)	0.0209 (7)	0.0011 (6)	0.0061 (6)	0.0008 (6)
C1	0.0182 (9)	0.0300 (11)	0.0267 (10)	0.0034 (8)	0.0024 (7)	0.0024 (8)
C2	0.0193 (9)	0.0319 (11)	0.0244 (9)	0.0044 (8)	0.0086 (7)	0.0033 (8)
C3	0.0221 (9)	0.0255 (10)	0.0250 (9)	−0.0026 (7)	0.0075 (7)	0.0020 (8)
C4	0.0182 (8)	0.0285 (10)	0.0250 (9)	0.0022 (7)	0.0064 (7)	0.0022 (8)
C5	0.0209 (9)	0.0396 (13)	0.0391 (12)	0.0005 (9)	0.0112 (9)	0.0043 (10)
C6	0.0243 (9)	0.0269 (10)	0.0219 (9)	0.0073 (8)	0.0065 (7)	0.0052 (7)
C7	0.0257 (9)	0.0226 (10)	0.0227 (9)	0.0045 (7)	0.0080 (7)	0.0028 (7)
C8	0.0350 (12)	0.0249 (11)	0.0407 (12)	0.0078 (9)	0.0098 (10)	0.0027 (9)
C9	0.0454 (14)	0.0228 (11)	0.0465 (14)	0.0013 (10)	0.0127 (11)	−0.0057 (10)
C10	0.0362 (12)	0.0288 (11)	0.0326 (11)	−0.0050 (9)	0.0103 (9)	−0.0065 (9)
C11	0.0256 (10)	0.0309 (11)	0.0219 (9)	0.0005 (8)	0.0070 (8)	−0.0029 (8)
C12	0.0245 (9)	0.0259 (10)	0.0163 (8)	0.0017 (7)	0.0082 (7)	0.0021 (7)
C13	0.0241 (9)	0.0233 (9)	0.0177 (8)	0.0004 (7)	0.0066 (7)	0.0009 (7)
C14	0.0207 (9)	0.0237 (10)	0.0197 (8)	0.0001 (7)	0.0040 (7)	0.0004 (7)
C15	0.0259 (10)	0.0340 (12)	0.0222 (9)	0.0043 (9)	0.0034 (8)	0.0039 (8)
C16	0.0268 (10)	0.0434 (13)	0.0320 (11)	0.0124 (10)	0.0014 (9)	0.0119 (10)
C17	0.0223 (10)	0.0388 (13)	0.0381 (12)	0.0098 (9)	0.0048 (9)	0.0055 (10)
C18	0.0196 (9)	0.0305 (11)	0.0271 (10)	0.0020 (8)	0.0055 (8)	0.0000 (8)
C19	0.0182 (8)	0.0193 (9)	0.0220 (9)	0.0001 (7)	0.0032 (7)	−0.0003 (7)
C20	0.0220 (9)	0.0193 (9)	0.0248 (9)	−0.0006 (7)	0.0040 (7)	−0.0011 (7)
C21	0.0226 (9)	0.0236 (10)	0.0182 (8)	0.0030 (7)	0.0028 (7)	0.0000 (7)
C22	0.0302 (10)	0.0260 (10)	0.0216 (9)	−0.0009 (8)	0.0035 (8)	−0.0050 (8)
C23	0.0355 (11)	0.0342 (12)	0.0181 (9)	0.0033 (9)	0.0068 (8)	−0.0019 (8)
C24	0.0360 (11)	0.0303 (11)	0.0230 (9)	0.0027 (9)	0.0125 (8)	0.0054 (8)
C25	0.0299 (10)	0.0269 (10)	0.0227 (9)	−0.0019 (8)	0.0067 (8)	0.0011 (8)
C26	0.0229 (9)	0.0222 (9)	0.0169 (8)	0.0042 (7)	0.0040 (7)	0.0005 (7)
Ga2	0.02359 (11)	0.02028 (11)	0.01746 (10)	−0.00167 (8)	0.00174 (8)	0.00285 (8)
O4	0.0273 (7)	0.0247 (7)	0.0197 (6)	−0.0027 (6)	0.0010 (5)	0.0043 (5)
O5	0.0307 (7)	0.0243 (7)	0.0240 (7)	−0.0059 (6)	0.0065 (6)	0.0007 (6)
O6	0.0330 (8)	0.0219 (7)	0.0208 (7)	0.0010 (6)	0.0070 (6)	0.0041 (5)
N4	0.0243 (8)	0.0221 (8)	0.0205 (7)	−0.0007 (6)	0.0005 (6)	0.0039 (6)
N5	0.0269 (8)	0.0253 (9)	0.0225 (8)	0.0005 (7)	0.0025 (7)	0.0041 (7)
N6	0.0309 (9)	0.0219 (8)	0.0203 (8)	−0.0020 (7)	0.0002 (7)	0.0043 (6)
C27	0.0245 (10)	0.0294 (11)	0.0304 (10)	−0.0034 (8)	−0.0029 (8)	0.0078 (9)
C28	0.0272 (10)	0.0314 (11)	0.0333 (11)	0.0072 (9)	0.0058 (9)	0.0068 (9)
C29	0.0354 (11)	0.0289 (11)	0.0256 (10)	0.0034 (9)	−0.0020 (9)	0.0082 (8)
C30	0.0277 (10)	0.0297 (11)	0.0287 (10)	0.0020 (8)	−0.0002 (8)	0.0074 (8)
C31	0.0287 (12)	0.0487 (16)	0.0496 (15)	0.0070 (11)	−0.0021 (11)	0.0179 (12)
C32	0.0217 (9)	0.0208 (9)	0.0243 (9)	0.0010 (7)	0.0065 (7)	0.0007 (7)
C33	0.0240 (9)	0.0217 (9)	0.0204 (8)	0.0048 (7)	0.0085 (7)	0.0039 (7)
C34	0.0299 (10)	0.0233 (10)	0.0282 (10)	0.0048 (8)	0.0117 (8)	0.0056 (8)
C35	0.0429 (12)	0.0231 (10)	0.0262 (10)	0.0100 (9)	0.0130 (9)	0.0087 (8)
C36	0.0377 (12)	0.0313 (11)	0.0217 (9)	0.0137 (9)	0.0053 (8)	0.0051 (8)
C37	0.0276 (10)	0.0310 (11)	0.0206 (9)	0.0058 (8)	0.0039 (8)	0.0017 (8)
C38	0.0249 (9)	0.0236 (10)	0.0188 (8)	0.0053 (8)	0.0075 (7)	0.0028 (7)
C39	0.0377 (12)	0.0262 (11)	0.0253 (10)	0.0050 (9)	0.0049 (9)	0.0014 (8)
C40	0.0383 (12)	0.0210 (10)	0.0246 (10)	−0.0021 (9)	0.0002 (9)	0.0025 (8)
C41	0.0516 (15)	0.0249 (11)	0.0337 (12)	0.0029 (10)	0.0020 (11)	−0.0040 (9)
C42	0.0551 (16)	0.0261 (12)	0.0451 (14)	−0.0064 (11)	−0.0065 (12)	−0.0036 (11)
C43	0.0412 (14)	0.0308 (13)	0.0498 (15)	−0.0109 (11)	−0.0094 (12)	0.0030 (11)
C44	0.0336 (12)	0.0290 (12)	0.0423 (13)	−0.0062 (9)	0.0021 (10)	0.0055 (10)
C45	0.0339 (11)	0.0192 (9)	0.0238 (9)	−0.0036 (8)	0.0002 (8)	0.0055 (7)
C46	0.0391 (12)	0.0243 (10)	0.0189 (9)	−0.0030 (9)	0.0008 (8)	0.0051 (7)
C47	0.0372 (11)	0.0229 (10)	0.0217 (9)	−0.0053 (8)	0.0061 (8)	0.0016 (7)
C48	0.0473 (13)	0.0290 (11)	0.0219 (10)	−0.0088 (10)	0.0083 (9)	0.0032 (8)
C49	0.0495 (14)	0.0363 (13)	0.0243 (10)	−0.0124 (11)	0.0156 (10)	−0.0017 (9)
C50	0.0356 (12)	0.0390 (13)	0.0325 (12)	−0.0082 (10)	0.0120 (10)	−0.0054 (10)
C51	0.0319 (11)	0.0347 (12)	0.0261 (10)	−0.0011 (9)	0.0058 (9)	−0.0001 (9)
C52	0.0298 (10)	0.0220 (10)	0.0190 (9)	−0.0084 (8)	0.0031 (7)	−0.0032 (7)
N7	0.123 (3)	0.0493 (18)	0.141 (3)	0.0348 (19)	0.077 (3)	0.0124 (19)
C53	0.090 (2)	0.0246 (13)	0.0566 (18)	0.0070 (15)	0.0214 (17)	−0.0010 (12)
C54	0.064 (2)	0.0500 (19)	0.092 (3)	0.0034 (16)	0.0055 (19)	−0.0236 (18)
N8	0.085 (4)	0.156 (8)	0.067 (4)	−0.042 (5)	0.024 (4)	−0.003 (4)
C55	0.101 (5)	0.062 (6)	0.031 (4)	−0.032 (5)	0.025 (5)	−0.005 (4)
C56	0.096 (6)	0.083 (9)	0.076 (7)	0.000 (7)	−0.021 (6)	0.044 (6)

### ({[(2,2-Bis{[(2-oxidobenzylidene)amino-κ2N,O]methyl}propyl)imino]methyl}phenololato-κ2N,O)gallium(III) acetonitrile 0.75-solvate (1b) Geometric parameters (Å, º)

**Table:**

Ga1—O3	1.9175 (13)	Ga2—N6	2.0984 (16)
Ga1—O1	1.9215 (13)	O4—C38	1.313 (2)
Ga1—O2	1.9302 (13)	O5—C45	1.316 (2)
Ga1—N1	2.0668 (16)	O6—C52	1.321 (2)
Ga1—N3	2.0719 (16)	N4—C32	1.288 (2)
Ga1—N2	2.0976 (16)	N4—C27	1.467 (2)
O1—C12	1.318 (2)	N5—C39	1.280 (3)
O2—C19	1.312 (2)	N5—C28	1.478 (3)
O3—C26	1.321 (2)	N6—C46	1.284 (3)
N1—C6	1.288 (2)	N6—C29	1.474 (3)
N1—C1	1.475 (2)	C27—C30	1.542 (3)
N2—C13	1.284 (2)	C27—H27A	0.9900
N2—C2	1.474 (2)	C27—H27B	0.9900
N3—C20	1.282 (2)	C28—C30	1.535 (3)
N3—C3	1.468 (2)	C28—H28A	0.9900
C1—C4	1.548 (3)	C28—H28B	0.9900
C1—H1A	0.9900	C29—C30	1.531 (3)
C1—H1B	0.9900	C29—H29A	0.9900
C2—C4	1.542 (3)	C29—H29B	0.9900
C2—H2A	0.9900	C30—C31	1.531 (3)
C2—H2B	0.9900	C31—H31A	0.9800
C3—C4	1.540 (3)	C31—H31B	0.9800
C3—H3A	0.9900	C31—H31C	0.9800
C3—H3B	0.9900	C32—C33	1.436 (3)
C4—C5	1.530 (3)	C32—H32	0.9500
C5—H5A	0.9800	C33—C34	1.408 (3)
C5—H5B	0.9800	C33—C38	1.418 (3)
C5—H5C	0.9800	C34—C35	1.372 (3)
C6—C7	1.447 (3)	C34—H34	0.9500
C6—H6	0.9500	C35—C36	1.394 (3)
C7—C8	1.409 (3)	C35—H35	0.9500
C7—C12	1.416 (3)	C36—C37	1.373 (3)
C8—C9	1.373 (3)	C36—H36	0.9500
C8—H8	0.9500	C37—C38	1.413 (3)
C9—C10	1.393 (3)	C37—H37	0.9500
C9—H9	0.9500	C39—C40	1.440 (3)
C10—C11	1.379 (3)	C39—H39	0.9500
C10—H10	0.9500	C40—C41	1.402 (3)
C11—C12	1.413 (3)	C40—C45	1.413 (3)
C11—H11	0.9500	C41—C42	1.374 (4)
C13—C14	1.434 (3)	C41—H41	0.9500
C13—H13	0.9500	C42—C43	1.390 (4)
C14—C15	1.408 (3)	C42—H42	0.9500
C14—C19	1.421 (3)	C43—C44	1.375 (3)
C15—C16	1.369 (3)	C43—H43	0.9500
C15—H15	0.9500	C44—C45	1.419 (3)
C16—C17	1.396 (3)	C44—H44	0.9500
C16—H16	0.9500	C46—C47	1.441 (3)
C17—C18	1.381 (3)	C46—H46	0.9500
C17—H17	0.9500	C47—C48	1.414 (3)
C18—C19	1.410 (3)	C47—C52	1.419 (3)
C18—H18	0.9500	C48—C49	1.364 (3)
C20—C21	1.436 (3)	C48—H48	0.9500
C20—H20	0.9500	C49—C50	1.396 (3)
C21—C22	1.413 (3)	C49—H49	0.9500
C21—C26	1.417 (3)	C50—C51	1.380 (3)
C22—C23	1.362 (3)	C50—H50	0.9500
C22—H22	0.9500	C51—C52	1.407 (3)
C23—C24	1.393 (3)	C51—H51	0.9500
C23—H23	0.9500	N7—C53	1.126 (4)
C24—C25	1.376 (3)	C53—C54	1.435 (4)
C24—H24	0.9500	C54—H54A	0.9800
C25—C26	1.409 (3)	C54—H54B	0.9800
C25—H25	0.9500	C54—H54C	0.9800
Ga2—O6	1.9238 (14)	N8—C55	1.138 (9)
Ga2—O5	1.9239 (14)	C55—C56	1.464 (7)
Ga2—O4	1.9296 (13)	C56—H56A	0.9800
Ga2—N4	2.0583 (16)	C56—H56B	0.9800
Ga2—N5	2.0897 (18)	C56—H56C	0.9800
			
O3—Ga1—O1	92.70 (6)	O5—Ga2—N5	87.99 (6)
O3—Ga1—O2	94.28 (6)	O4—Ga2—N5	97.10 (6)
O1—Ga1—O2	91.39 (6)	N4—Ga2—N5	84.35 (7)
O3—Ga1—N1	95.36 (6)	O6—Ga2—N6	87.38 (6)
O1—Ga1—N1	89.77 (6)	O5—Ga2—N6	93.86 (6)
O2—Ga1—N1	170.22 (6)	O4—Ga2—N6	174.83 (6)
O3—Ga1—N3	89.22 (6)	N4—Ga2—N6	85.69 (6)
O1—Ga1—N3	174.65 (6)	N5—Ga2—N6	82.12 (7)
O2—Ga1—N3	93.44 (6)	C38—O4—Ga2	128.73 (12)
N1—Ga1—N3	85.08 (6)	C45—O5—Ga2	126.13 (13)
O3—Ga1—N2	174.19 (6)	C52—O6—Ga2	124.41 (13)
O1—Ga1—N2	92.79 (6)	C32—N4—C27	116.98 (17)
O2—Ga1—N2	87.50 (6)	C32—N4—Ga2	125.53 (13)
N1—Ga1—N2	82.74 (6)	C27—N4—Ga2	117.33 (12)
N3—Ga1—N2	85.15 (6)	C39—N5—C28	117.77 (19)
C12—O1—Ga1	129.23 (12)	C39—N5—Ga2	124.47 (15)
C19—O2—Ga1	128.71 (12)	C28—N5—Ga2	117.73 (13)
C26—O3—Ga1	129.25 (12)	C46—N6—C29	118.37 (18)
C6—N1—C1	117.43 (17)	C46—N6—Ga2	121.62 (15)
C6—N1—Ga1	125.09 (13)	C29—N6—Ga2	119.60 (13)
C1—N1—Ga1	117.36 (13)	N4—C27—C30	110.78 (16)
C13—N2—C2	117.85 (16)	N4—C27—H27A	109.5
C13—N2—Ga1	124.19 (13)	C30—C27—H27A	109.5
C2—N2—Ga1	117.90 (12)	N4—C27—H27B	109.5
C20—N3—C3	117.81 (16)	C30—C27—H27B	109.5
C20—N3—Ga1	125.21 (14)	H27A—C27—H27B	108.1
C3—N3—Ga1	116.74 (12)	N5—C28—C30	110.71 (17)
N1—C1—C4	109.32 (15)	N5—C28—H28A	109.5
N1—C1—H1A	109.8	C30—C28—H28A	109.5
C4—C1—H1A	109.8	N5—C28—H28B	109.5
N1—C1—H1B	109.8	C30—C28—H28B	109.5
C4—C1—H1B	109.8	H28A—C28—H28B	108.1
H1A—C1—H1B	108.3	N6—C29—C30	110.29 (17)
N2—C2—C4	109.46 (15)	N6—C29—H29A	109.6
N2—C2—H2A	109.8	C30—C29—H29A	109.6
C4—C2—H2A	109.8	N6—C29—H29B	109.6
N2—C2—H2B	109.8	C30—C29—H29B	109.6
C4—C2—H2B	109.8	H29A—C29—H29B	108.1
H2A—C2—H2B	108.2	C31—C30—C29	110.19 (18)
N3—C3—C4	110.14 (16)	C31—C30—C28	108.7 (2)
N3—C3—H3A	109.6	C29—C30—C28	109.88 (17)
C4—C3—H3A	109.6	C31—C30—C27	107.98 (18)
N3—C3—H3B	109.6	C29—C30—C27	109.15 (19)
C4—C3—H3B	109.6	C28—C30—C27	110.96 (17)
H3A—C3—H3B	108.1	C30—C31—H31A	109.5
C5—C4—C3	107.96 (17)	C30—C31—H31B	109.5
C5—C4—C2	109.08 (16)	H31A—C31—H31B	109.5
C3—C4—C2	109.90 (16)	C30—C31—H31C	109.5
C5—C4—C1	109.85 (17)	H31A—C31—H31C	109.5
C3—C4—C1	110.62 (16)	H31B—C31—H31C	109.5
C2—C4—C1	109.40 (16)	N4—C32—C33	125.58 (18)
C4—C5—H5A	109.5	N4—C32—H32	117.2
C4—C5—H5B	109.5	C33—C32—H32	117.2
H5A—C5—H5B	109.5	C34—C33—C38	120.46 (18)
C4—C5—H5C	109.5	C34—C33—C32	116.57 (18)
H5A—C5—H5C	109.5	C38—C33—C32	122.89 (17)
H5B—C5—H5C	109.5	C35—C34—C33	121.1 (2)
N1—C6—C7	125.26 (18)	C35—C34—H34	119.5
N1—C6—H6	117.4	C33—C34—H34	119.5
C7—C6—H6	117.4	C34—C35—C36	118.82 (19)
C8—C7—C12	119.92 (19)	C34—C35—H35	120.6
C8—C7—C6	116.42 (18)	C36—C35—H35	120.6
C12—C7—C6	123.18 (18)	C37—C36—C35	121.33 (19)
C9—C8—C7	121.5 (2)	C37—C36—H36	119.3
C9—C8—H8	119.3	C35—C36—H36	119.3
C7—C8—H8	119.3	C36—C37—C38	121.5 (2)
C8—C9—C10	118.9 (2)	C36—C37—H37	119.3
C8—C9—H9	120.6	C38—C37—H37	119.3
C10—C9—H9	120.6	O4—C38—C37	119.25 (18)
C11—C10—C9	121.1 (2)	O4—C38—C33	123.93 (17)
C11—C10—H10	119.5	C37—C38—C33	116.80 (18)
C9—C10—H10	119.5	N5—C39—C40	125.3 (2)
C10—C11—C12	121.3 (2)	N5—C39—H39	117.3
C10—C11—H11	119.3	C40—C39—H39	117.3
C12—C11—H11	119.3	C41—C40—C45	119.9 (2)
O1—C12—C11	118.23 (18)	C41—C40—C39	117.7 (2)
O1—C12—C7	124.37 (17)	C45—C40—C39	122.34 (19)
C11—C12—C7	117.34 (18)	C42—C41—C40	121.5 (2)
N2—C13—C14	125.08 (17)	C42—C41—H41	119.3
N2—C13—H13	117.5	C40—C41—H41	119.3
C14—C13—H13	117.5	C41—C42—C43	118.9 (2)
C15—C14—C19	119.84 (18)	C41—C42—H42	120.5
C15—C14—C13	116.94 (17)	C43—C42—H42	120.5
C19—C14—C13	122.51 (17)	C44—C43—C42	121.2 (2)
C16—C15—C14	122.03 (19)	C44—C43—H43	119.4
C16—C15—H15	119.0	C42—C43—H43	119.4
C14—C15—H15	119.0	C43—C44—C45	121.0 (2)
C15—C16—C17	118.4 (2)	C43—C44—H44	119.5
C15—C16—H16	120.8	C45—C44—H44	119.5
C17—C16—H16	120.8	O5—C45—C40	123.83 (19)
C18—C17—C16	121.2 (2)	O5—C45—C44	118.7 (2)
C18—C17—H17	119.4	C40—C45—C44	117.4 (2)
C16—C17—H17	119.4	N6—C46—C47	125.52 (19)
C17—C18—C19	121.59 (19)	N6—C46—H46	117.2
C17—C18—H18	119.2	C47—C46—H46	117.2
C19—C18—H18	119.2	C48—C47—C52	119.5 (2)
O2—C19—C18	118.49 (17)	C48—C47—C46	117.3 (2)
O2—C19—C14	124.48 (17)	C52—C47—C46	123.03 (19)
C18—C19—C14	116.94 (18)	C49—C48—C47	121.5 (2)
N3—C20—C21	126.00 (18)	C49—C48—H48	119.3
N3—C20—H20	117.0	C47—C48—H48	119.3
C21—C20—H20	117.0	C48—C49—C50	119.2 (2)
C22—C21—C26	119.79 (18)	C48—C49—H49	120.4
C22—C21—C20	117.58 (17)	C50—C49—H49	120.4
C26—C21—C20	122.42 (17)	C51—C50—C49	120.9 (2)
C23—C22—C21	121.60 (19)	C51—C50—H50	119.5
C23—C22—H22	119.2	C49—C50—H50	119.5
C21—C22—H22	119.2	C50—C51—C52	121.2 (2)
C22—C23—C24	118.88 (18)	C50—C51—H51	119.4
C22—C23—H23	120.6	C52—C51—H51	119.4
C24—C23—H23	120.6	O6—C52—C51	119.52 (19)
C25—C24—C23	121.10 (19)	O6—C52—C47	122.7 (2)
C25—C24—H24	119.5	C51—C52—C47	117.70 (19)
C23—C24—H24	119.5	N7—C53—C54	179.8 (4)
C24—C25—C26	121.47 (19)	C53—C54—H54A	109.5
C24—C25—H25	119.3	C53—C54—H54B	109.5
C26—C25—H25	119.3	H54A—C54—H54B	109.5
O3—C26—C25	118.51 (17)	C53—C54—H54C	109.5
O3—C26—C21	124.38 (17)	H54A—C54—H54C	109.5
C25—C26—C21	117.09 (17)	H54B—C54—H54C	109.5
O6—Ga2—O5	91.00 (6)	N8—C55—C56	173.9 (10)
O6—Ga2—O4	93.51 (6)	C55—C56—H56A	109.5
O5—Ga2—O4	91.22 (6)	C55—C56—H56B	109.5
O6—Ga2—N4	96.64 (6)	H56A—C56—H56B	109.5
O5—Ga2—N4	172.32 (7)	C55—C56—H56C	109.5
O4—Ga2—N4	89.15 (6)	H56A—C56—H56C	109.5
O6—Ga2—N5	169.36 (6)	H56B—C56—H56C	109.5
			
C6—N1—C1—C4	−134.82 (18)	C32—N4—C27—C30	134.21 (19)
Ga1—N1—C1—C4	41.39 (19)	Ga2—N4—C27—C30	−41.6 (2)
C13—N2—C2—C4	−144.97 (18)	C39—N5—C28—C30	140.67 (19)
Ga1—N2—C2—C4	37.7 (2)	Ga2—N5—C28—C30	−37.6 (2)
C20—N3—C3—C4	−132.89 (18)	C46—N6—C29—C30	154.60 (18)
Ga1—N3—C3—C4	41.7 (2)	Ga2—N6—C29—C30	−32.7 (2)
N3—C3—C4—C5	155.31 (17)	N6—C29—C30—C31	−159.72 (19)
N3—C3—C4—C2	−85.82 (19)	N6—C29—C30—C28	80.6 (2)
N3—C3—C4—C1	35.1 (2)	N6—C29—C30—C27	−41.3 (2)
N2—C2—C4—C5	156.62 (17)	N5—C28—C30—C31	−161.28 (18)
N2—C2—C4—C3	38.4 (2)	N5—C28—C30—C29	−40.7 (2)
N2—C2—C4—C1	−83.20 (19)	N5—C28—C30—C27	80.2 (2)
N1—C1—C4—C5	157.89 (17)	N4—C27—C30—C31	−154.34 (19)
N1—C1—C4—C3	−83.03 (19)	N4—C27—C30—C29	85.9 (2)
N1—C1—C4—C2	38.2 (2)	N4—C27—C30—C28	−35.4 (2)
C1—N1—C6—C7	168.79 (18)	C27—N4—C32—C33	−174.73 (19)
Ga1—N1—C6—C7	−7.1 (3)	Ga2—N4—C32—C33	0.6 (3)
N1—C6—C7—C8	−179.54 (19)	N4—C32—C33—C34	−172.83 (19)
N1—C6—C7—C12	−7.5 (3)	N4—C32—C33—C38	10.3 (3)
C12—C7—C8—C9	−0.3 (3)	C38—C33—C34—C35	−1.9 (3)
C6—C7—C8—C9	172.0 (2)	C32—C33—C34—C35	−178.84 (19)
C7—C8—C9—C10	−0.9 (4)	C33—C34—C35—C36	1.4 (3)
C8—C9—C10—C11	1.0 (4)	C34—C35—C36—C37	0.2 (3)
C9—C10—C11—C12	0.2 (3)	C35—C36—C37—C38	−1.3 (3)
Ga1—O1—C12—C11	−173.14 (13)	Ga2—O4—C38—C37	162.04 (14)
Ga1—O1—C12—C7	9.6 (3)	Ga2—O4—C38—C33	−19.5 (3)
C10—C11—C12—O1	−178.80 (18)	C36—C37—C38—O4	179.43 (18)
C10—C11—C12—C7	−1.4 (3)	C36—C37—C38—C33	0.8 (3)
C8—C7—C12—O1	178.68 (18)	C34—C33—C38—O4	−177.79 (18)
C6—C7—C12—O1	6.9 (3)	C32—C33—C38—O4	−1.0 (3)
C8—C7—C12—C11	1.4 (3)	C34—C33—C38—C37	0.7 (3)
C6—C7—C12—C11	−170.34 (17)	C32—C33—C38—C37	177.52 (18)
C2—N2—C13—C14	167.67 (18)	C28—N5—C39—C40	−178.1 (2)
Ga1—N2—C13—C14	−15.2 (3)	Ga2—N5—C39—C40	0.1 (3)
N2—C13—C14—C15	−177.88 (19)	N5—C39—C40—C41	−165.2 (2)
N2—C13—C14—C19	−7.6 (3)	N5—C39—C40—C45	14.7 (3)
C19—C14—C15—C16	−1.6 (3)	C45—C40—C41—C42	−1.2 (3)
C13—C14—C15—C16	169.0 (2)	C39—C40—C41—C42	178.7 (2)
C14—C15—C16—C17	−1.0 (3)	C40—C41—C42—C43	2.9 (4)
C15—C16—C17—C18	2.2 (3)	C41—C42—C43—C44	−1.2 (4)
C16—C17—C18—C19	−0.8 (3)	C42—C43—C44—C45	−2.2 (4)
Ga1—O2—C19—C18	−169.20 (13)	Ga2—O5—C45—C40	−31.3 (3)
Ga1—O2—C19—C14	14.2 (3)	Ga2—O5—C45—C44	151.55 (16)
C17—C18—C19—O2	−178.59 (19)	C41—C40—C45—O5	−179.26 (19)
C17—C18—C19—C14	−1.7 (3)	C39—C40—C45—O5	0.9 (3)
C15—C14—C19—O2	179.53 (18)	C41—C40—C45—C44	−2.1 (3)
C13—C14—C19—O2	9.5 (3)	C39—C40—C45—C44	178.0 (2)
C15—C14—C19—C18	2.9 (3)	C43—C44—C45—O5	−178.9 (2)
C13—C14—C19—C18	−167.11 (18)	C43—C44—C45—C40	3.8 (3)
C3—N3—C20—C21	171.62 (19)	C29—N6—C46—C47	−174.31 (19)
Ga1—N3—C20—C21	−2.5 (3)	Ga2—N6—C46—C47	13.2 (3)
N3—C20—C21—C22	174.4 (2)	N6—C46—C47—C48	−173.7 (2)
N3—C20—C21—C26	−10.9 (3)	N6—C46—C47—C52	10.2 (3)
C26—C21—C22—C23	0.0 (3)	C52—C47—C48—C49	−1.1 (3)
C20—C21—C22—C23	174.9 (2)	C46—C47—C48—C49	−177.4 (2)
C21—C22—C23—C24	−1.3 (3)	C47—C48—C49—C50	1.5 (3)
C22—C23—C24—C25	0.3 (3)	C48—C49—C50—C51	−0.5 (3)
C23—C24—C25—C26	2.0 (3)	C49—C50—C51—C52	−0.8 (3)
Ga1—O3—C26—C25	−168.30 (14)	Ga2—O6—C52—C51	149.73 (15)
Ga1—O3—C26—C21	13.5 (3)	Ga2—O6—C52—C47	−33.5 (2)
C24—C25—C26—O3	178.51 (19)	C50—C51—C52—O6	178.01 (18)
C24—C25—C26—C21	−3.2 (3)	C50—C51—C52—C47	1.1 (3)
C22—C21—C26—O3	−179.63 (18)	C48—C47—C52—O6	−176.98 (18)
C20—C21—C26—O3	5.8 (3)	C46—C47—C52—O6	−0.9 (3)
C22—C21—C26—C25	2.2 (3)	C48—C47—C52—C51	−0.2 (3)
C20—C21—C26—C25	−172.45 (19)	C46—C47—C52—C51	175.84 (19)

### ({[(2,2-Bis{[(2-oxidobenzylidene)amino-κ2N,O]methyl}propyl)imino]methyl}phenololato-κ2N,O)gallium(III) acetonitrile 0.75-solvate (1b) Hydrogen-bond geometry (Å, º)

**Table:**

*D*—H···*A*	*D*—H	H···*A*	*D*···*A*	*D*—H···*A*
C32—H32···O1	0.95	2.51	3.333 (2)	146
C34—H34···O1	0.95	2.88	3.574 (2)	131
C15—H15···O1^i^	0.95	2.65	3.499 (2)	149
C24—H24···O2^ii^	0.95	2.83	3.610 (2)	140
C54—H54*B*···O2^iii^	0.98	2.31	3.282 (3)	171
C27—H27*A*···O3	0.99	2.89	3.697 (2)	140
C6—H6···O4^iv^	0.95	2.68	3.557 (2)	153
C8—H8···O4^iv^	0.95	2.84	3.642 (3)	143
C8—H8···O5^iv^	0.95	2.91	3.806 (3)	157
C48—H48···O5^v^	0.95	2.55	3.413 (3)	151
C6—H6···O6^iv^	0.95	2.54	3.325 (2)	140
C22—H22···O6^ii^	0.95	2.56	3.502 (2)	173
C28—H28*A*···N7	0.99	2.91	3.680 (4)	135
C29—H29*B*···N7	0.99	2.72	3.554 (4)	143
C31—H31*C*···N7	0.98	2.85	3.703 (4)	146
C10—H10···N8^vi^	0.95	2.63	3.508 (7)	154

Symmetry codes: (i) −*x*+1, −*y*, −*z*+1; (ii) −*x*+1, −*y*, −*z*; (iii) *x*, *y*+1, *z*; (iv) *x*−1, *y*, *z*; (v) −*x*+2, −*y*+1, −*z*; (vi) −*x*+1, −*y*+1, −*z*+1.

### ({[(2,2-Bis{[(2-oxidobenzylidene)amino-κ2N,O]methyl}propyl)imino]methyl}phenololato-κ2N,O)indium(III) dichloromethane monosolvate (2) Crystal data

**Table:**

[In(C_26_H_24_N_3_O_3_)]·CH_2_Cl_2_	*F*(000) = 1264
*M**_r_* = 626.23	*D*_x_ = 1.591 Mg m^−^^3^
Monoclinic, *P*2_1_/*c*	Mo *K*α radiation, λ = 0.71073 Å
*a* = 10.0704 (2) Å	Cell parameters from 17154 reflections
*b* = 16.2514 (4) Å	θ = 2.4–32.9°
*c* = 16.1749 (4) Å	µ = 1.14 mm^−^^1^
β = 99.130 (2)°	*T* = 100 K
*V* = 2613.62 (11) Å^3^	Needle, colourless
*Z* = 4	0.34 × 0.14 × 0.07 mm

### ({[(2,2-Bis{[(2-oxidobenzylidene)amino-κ2N,O]methyl}propyl)imino]methyl}phenololato-κ2N,O)indium(III) dichloromethane monosolvate (2) Data collection

**Table:**

XtaLAB Synergy, Dualflex, HyPix diffractometer	8621 independent reflections
Radiation source: micro-focus sealed X-ray tube, PhotonJet (Mo) X-ray Source	7401 reflections with *I* > 2σ(*I*)
Mirror monochromator	*R*_int_ = 0.037
Detector resolution: 10.0000 pixels mm^-1^	θ_max_ = 33.1°, θ_min_ = 2.5°
ω scans	*h* = −15→13
Absorption correction: multi-scan (CrysAlisPro; Rigaku OD, 2019)	*k* = −21→24
*T*_min_ = 0.676, *T*_max_ = 1.000	*l* = −22→24
31229 measured reflections	

### ({[(2,2-Bis{[(2-oxidobenzylidene)amino-κ2N,O]methyl}propyl)imino]methyl}phenololato-κ2N,O)indium(III) dichloromethane monosolvate (2) Refinement

**Table:**

Refinement on *F*^2^	Primary atom site location: dual
Least-squares matrix: full	Secondary atom site location: difference Fourier map
*R*[*F*^2^ > 2σ(*F*^2^)] = 0.029	Hydrogen site location: inferred from neighbouring sites
*wR*(*F*^2^) = 0.064	H-atom parameters constrained
*S* = 1.06	*w* = 1/[σ^2^(*F*_o_^2^) + (0.0232*P*)^2^ + 0.8708*P*] where *P* = (*F*_o_^2^ + 2*F*_c_^2^)/3
8621 reflections	(Δ/σ)_max_ = 0.003
326 parameters	Δρ_max_ = 0.62 e Å^−^^3^
0 restraints	Δρ_min_ = −0.53 e Å^−^^3^

### ({[(2,2-Bis{[(2-oxidobenzylidene)amino-κ2N,O]methyl}propyl)imino]methyl}phenololato-κ2N,O)indium(III) dichloromethane monosolvate (2) Special details

**Table:**

Geometry. All esds (except the esd in the dihedral angle between two l.s. planes) are estimated using the full covariance matrix. The cell esds are taken into account individually in the estimation of esds in distances, angles and torsion angles; correlations between esds in cell parameters are only used when they are defined by crystal symmetry. An approximate (isotropic) treatment of cell esds is used for estimating esds involving l.s. planes.

### ({[(2,2-Bis{[(2-oxidobenzylidene)amino-κ2N,O]methyl}propyl)imino]methyl}phenololato-κ2N,O)indium(III) dichloromethane monosolvate (2) Fractional atomic coordinates and isotropic or equivalent isotropic displacement parameters (Å^2^)

**Table:**

	*x*	*y*	*z*	*U*_iso_*/*U*_eq_	
In1	0.44234 (2)	0.76734 (2)	0.60863 (2)	0.01380 (4)	
O1	0.30272 (11)	0.85701 (7)	0.63247 (7)	0.0179 (2)	
O2	0.40058 (12)	0.78742 (7)	0.47927 (7)	0.0192 (2)	
O3	0.29061 (11)	0.67774 (7)	0.60288 (8)	0.0194 (2)	
N1	0.51813 (14)	0.77671 (8)	0.74596 (8)	0.0165 (3)	
N2	0.63141 (13)	0.83756 (8)	0.59915 (8)	0.0168 (3)	
N3	0.58328 (13)	0.65939 (8)	0.62197 (8)	0.0161 (3)	
C1	0.65051 (17)	0.73976 (10)	0.77773 (10)	0.0196 (3)	
H1A	0.638433	0.682044	0.789904	0.024*	
H1B	0.688637	0.766753	0.829572	0.024*	
C2	0.73966 (16)	0.83319 (10)	0.67196 (10)	0.0196 (3)	
H2A	0.723603	0.874388	0.712624	0.024*	
H2B	0.824759	0.845933	0.653984	0.024*	
C3	0.72684 (15)	0.67764 (10)	0.64906 (11)	0.0195 (3)	
H3A	0.766964	0.693095	0.600612	0.023*	
H3B	0.771881	0.628327	0.672869	0.023*	
C4	0.74959 (16)	0.74748 (10)	0.71434 (11)	0.0183 (3)	
C5	0.89334 (18)	0.73819 (11)	0.76248 (12)	0.0250 (4)	
H5A	0.955679	0.735006	0.723426	0.038*	
H5B	0.899158	0.688889	0.795556	0.038*	
H5C	0.914876	0.784863	0.798484	0.038*	
C6	0.44813 (16)	0.80394 (10)	0.79995 (10)	0.0174 (3)	
H6	0.483528	0.795509	0.855990	0.021*	
C7	0.31986 (16)	0.84640 (10)	0.78311 (10)	0.0168 (3)	
C8	0.26184 (17)	0.86798 (11)	0.85429 (11)	0.0228 (3)	
H8	0.301662	0.849411	0.906786	0.027*	
C9	0.14814 (18)	0.91570 (12)	0.84762 (12)	0.0282 (4)	
H9	0.112293	0.929965	0.895214	0.034*	
C10	0.08675 (18)	0.94264 (12)	0.76874 (12)	0.0274 (4)	
H10	0.009923	0.975165	0.763955	0.033*	
C11	0.13927 (17)	0.92134 (11)	0.69770 (11)	0.0225 (3)	
H11	0.096553	0.939458	0.645680	0.027*	
C12	0.25668 (16)	0.87255 (10)	0.70252 (10)	0.0173 (3)	
C13	0.64839 (16)	0.88497 (10)	0.53775 (10)	0.0179 (3)	
H13	0.729159	0.913720	0.543284	0.021*	
C14	0.55527 (16)	0.89815 (10)	0.46131 (10)	0.0170 (3)	
C15	0.58775 (17)	0.96171 (10)	0.40835 (10)	0.0208 (3)	
H15	0.666516	0.991607	0.423808	0.025*	
C16	0.50515 (18)	0.98046 (10)	0.33410 (10)	0.0223 (3)	
H16	0.528051	1.022498	0.299972	0.027*	
C17	0.38735 (18)	0.93571 (11)	0.31103 (10)	0.0219 (3)	
H17	0.330829	0.948394	0.261440	0.026*	
C18	0.35340 (17)	0.87253 (10)	0.36106 (10)	0.0193 (3)	
H18	0.274314	0.843322	0.344230	0.023*	
C19	0.43559 (15)	0.85131 (10)	0.43689 (9)	0.0159 (3)	
C20	0.54875 (15)	0.58334 (10)	0.61401 (10)	0.0160 (3)	
H20	0.617996	0.544907	0.622622	0.019*	
C21	0.41363 (15)	0.55107 (10)	0.59307 (9)	0.0151 (3)	
C22	0.40479 (16)	0.46536 (10)	0.57930 (10)	0.0190 (3)	
H22	0.483626	0.435241	0.580611	0.023*	
C23	0.28335 (17)	0.42487 (10)	0.56399 (11)	0.0219 (3)	
H23	0.279604	0.368547	0.553733	0.026*	
C24	0.16602 (16)	0.47043 (11)	0.56423 (10)	0.0208 (3)	
H24	0.083232	0.443813	0.555089	0.025*	
C25	0.17059 (16)	0.55445 (10)	0.57782 (10)	0.0191 (3)	
H25	0.090731	0.583086	0.578182	0.023*	
C26	0.29404 (15)	0.59796 (10)	0.59120 (9)	0.0154 (3)	
Cl1	0.03175 (6)	0.90823 (3)	0.44842 (3)	0.03845 (12)	
Cl2	−0.06104 (5)	0.74882 (3)	0.49805 (4)	0.03799 (12)	
C27	0.07878 (16)	0.80653 (11)	0.47822 (12)	0.0239 (4)	
H27A	0.118092	0.780353	0.433928	0.029*	
H27B	0.146259	0.807633	0.528180	0.029*	

### ({[(2,2-Bis{[(2-oxidobenzylidene)amino-κ2N,O]methyl}propyl)imino]methyl}phenololato-κ2N,O)indium(III) dichloromethane monosolvate (2) Atomic displacement parameters (Å^2^)

**Table:**

	*U*^11^	*U*^22^	*U*^33^	*U*^12^	*U*^13^	*U*^23^
In1	0.01471 (6)	0.01451 (6)	0.01177 (5)	−0.00124 (4)	0.00086 (4)	−0.00034 (4)
O1	0.0219 (5)	0.0169 (5)	0.0149 (5)	0.0031 (4)	0.0029 (4)	0.0005 (4)
O2	0.0230 (6)	0.0218 (6)	0.0123 (5)	−0.0078 (5)	0.0012 (4)	−0.0006 (4)
O3	0.0149 (5)	0.0168 (6)	0.0264 (6)	−0.0005 (4)	0.0030 (4)	−0.0010 (5)
N1	0.0185 (6)	0.0164 (6)	0.0140 (6)	−0.0020 (5)	0.0012 (5)	0.0012 (5)
N2	0.0182 (6)	0.0156 (6)	0.0154 (6)	−0.0023 (5)	−0.0002 (5)	−0.0012 (5)
N3	0.0138 (6)	0.0177 (6)	0.0170 (6)	−0.0022 (5)	0.0030 (5)	−0.0025 (5)
C1	0.0215 (8)	0.0204 (8)	0.0150 (7)	0.0015 (6)	−0.0027 (6)	0.0019 (6)
C2	0.0189 (7)	0.0198 (8)	0.0184 (8)	−0.0044 (6)	−0.0023 (6)	0.0000 (6)
C3	0.0122 (7)	0.0207 (8)	0.0253 (8)	−0.0018 (6)	0.0016 (6)	−0.0007 (6)
C4	0.0164 (7)	0.0179 (7)	0.0190 (8)	−0.0021 (6)	−0.0022 (6)	0.0004 (6)
C5	0.0200 (8)	0.0253 (9)	0.0270 (9)	−0.0036 (7)	−0.0049 (7)	0.0019 (7)
C6	0.0232 (8)	0.0159 (7)	0.0126 (7)	−0.0067 (6)	0.0012 (6)	0.0010 (6)
C7	0.0188 (7)	0.0161 (7)	0.0156 (7)	−0.0059 (6)	0.0036 (6)	−0.0006 (6)
C8	0.0222 (8)	0.0287 (9)	0.0180 (8)	−0.0089 (7)	0.0051 (6)	−0.0019 (7)
C9	0.0229 (8)	0.0380 (11)	0.0263 (9)	−0.0056 (8)	0.0117 (7)	−0.0083 (8)
C10	0.0190 (8)	0.0322 (10)	0.0317 (10)	0.0003 (7)	0.0064 (7)	−0.0065 (8)
C11	0.0215 (8)	0.0232 (8)	0.0228 (8)	0.0006 (7)	0.0031 (7)	−0.0015 (7)
C12	0.0195 (7)	0.0138 (7)	0.0188 (8)	−0.0043 (6)	0.0035 (6)	−0.0011 (6)
C13	0.0177 (7)	0.0159 (7)	0.0205 (8)	−0.0028 (6)	0.0045 (6)	−0.0024 (6)
C14	0.0204 (7)	0.0153 (7)	0.0158 (7)	−0.0001 (6)	0.0043 (6)	−0.0014 (6)
C15	0.0249 (8)	0.0172 (8)	0.0205 (8)	−0.0037 (6)	0.0039 (6)	−0.0011 (6)
C16	0.0333 (9)	0.0165 (8)	0.0179 (8)	−0.0006 (7)	0.0060 (7)	0.0027 (6)
C17	0.0298 (9)	0.0218 (8)	0.0140 (7)	0.0040 (7)	0.0036 (6)	−0.0004 (6)
C18	0.0210 (8)	0.0220 (8)	0.0147 (7)	−0.0011 (6)	0.0024 (6)	−0.0034 (6)
C19	0.0194 (7)	0.0160 (7)	0.0133 (7)	0.0011 (6)	0.0053 (6)	−0.0026 (5)
C20	0.0154 (7)	0.0167 (7)	0.0163 (7)	0.0008 (6)	0.0031 (6)	−0.0004 (6)
C21	0.0167 (7)	0.0163 (7)	0.0122 (7)	−0.0024 (6)	0.0024 (5)	0.0010 (5)
C22	0.0190 (7)	0.0177 (8)	0.0195 (8)	−0.0005 (6)	0.0007 (6)	0.0005 (6)
C23	0.0253 (8)	0.0159 (8)	0.0235 (8)	−0.0057 (7)	0.0010 (7)	0.0017 (6)
C24	0.0179 (7)	0.0229 (8)	0.0206 (8)	−0.0070 (6)	0.0006 (6)	0.0030 (6)
C25	0.0153 (7)	0.0224 (8)	0.0193 (8)	−0.0022 (6)	0.0020 (6)	0.0018 (6)
C26	0.0167 (7)	0.0180 (7)	0.0114 (7)	−0.0028 (6)	0.0023 (5)	0.0017 (5)
Cl1	0.0511 (3)	0.0208 (2)	0.0417 (3)	0.0032 (2)	0.0019 (2)	−0.00014 (19)
Cl2	0.0281 (2)	0.0428 (3)	0.0453 (3)	−0.0093 (2)	0.0126 (2)	0.0067 (2)
C27	0.0167 (7)	0.0207 (8)	0.0340 (10)	−0.0018 (6)	0.0030 (7)	−0.0023 (7)

### ({[(2,2-Bis{[(2-oxidobenzylidene)amino-κ2N,O]methyl}propyl)imino]methyl}phenololato-κ2N,O)indium(III) dichloromethane monosolvate (2) Geometric parameters (Å, º)

**Table:**

In1—O1	2.1027 (11)	C9—H9	0.9300
In1—O2	2.0935 (11)	C9—C10	1.397 (3)
In1—O3	2.1020 (11)	C10—H10	0.9300
In1—N1	2.2365 (14)	C10—C11	1.383 (2)
In1—N2	2.2458 (13)	C11—H11	0.9300
In1—N3	2.2453 (13)	C11—C12	1.416 (2)
O1—C12	1.3151 (19)	C13—H13	0.9300
O2—C19	1.3224 (19)	C13—C14	1.444 (2)
O3—C26	1.3116 (19)	C14—C15	1.413 (2)
N1—C1	1.478 (2)	C14—C19	1.427 (2)
N1—C6	1.285 (2)	C15—H15	0.9300
N2—C2	1.474 (2)	C15—C16	1.382 (2)
N2—C13	1.290 (2)	C16—H16	0.9300
N3—C3	1.473 (2)	C16—C17	1.391 (3)
N3—C20	1.285 (2)	C17—H17	0.9300
C1—H1A	0.9700	C17—C18	1.383 (2)
C1—H1B	0.9700	C18—H18	0.9300
C1—C4	1.545 (2)	C18—C19	1.409 (2)
C2—H2A	0.9700	C20—H20	0.9300
C2—H2B	0.9700	C20—C21	1.447 (2)
C2—C4	1.549 (2)	C21—C22	1.411 (2)
C3—H3A	0.9700	C21—C26	1.421 (2)
C3—H3B	0.9700	C22—H22	0.9300
C3—C4	1.542 (2)	C22—C23	1.376 (2)
C4—C5	1.538 (2)	C23—H23	0.9300
C5—H5A	0.9600	C23—C24	1.395 (2)
C5—H5B	0.9600	C24—H24	0.9300
C5—H5C	0.9600	C24—C25	1.383 (2)
C6—H6	0.9300	C25—H25	0.9300
C6—C7	1.452 (2)	C25—C26	1.417 (2)
C7—C8	1.415 (2)	Cl1—C27	1.7651 (18)
C7—C12	1.422 (2)	Cl2—C27	1.7631 (17)
C8—H8	0.9300	C27—H27A	0.9700
C8—C9	1.373 (3)	C27—H27B	0.9700
			
O1—In1—N1	84.46 (5)	C7—C8—H8	119.2
O1—In1—N2	105.02 (5)	C9—C8—C7	121.52 (17)
O1—In1—N3	162.70 (5)	C9—C8—H8	119.2
O2—In1—O1	92.35 (5)	C8—C9—H9	120.3
O2—In1—O3	91.97 (5)	C8—C9—C10	119.44 (16)
O2—In1—N1	164.77 (5)	C10—C9—H9	120.3
O2—In1—N2	83.71 (5)	C9—C10—H10	119.7
O2—In1—N3	103.97 (5)	C11—C10—C9	120.54 (17)
O3—In1—O1	89.18 (4)	C11—C10—H10	119.7
O3—In1—N1	102.84 (5)	C10—C11—H11	119.3
O3—In1—N2	165.28 (5)	C10—C11—C12	121.38 (17)
O3—In1—N3	84.66 (5)	C12—C11—H11	119.3
N1—In1—N2	82.75 (5)	O1—C12—C7	124.40 (15)
N1—In1—N3	81.19 (5)	O1—C12—C11	117.76 (15)
N3—In1—N2	82.75 (5)	C11—C12—C7	117.79 (15)
C12—O1—In1	128.94 (10)	N2—C13—H13	116.5
C19—O2—In1	127.79 (10)	N2—C13—C14	126.90 (15)
C26—O3—In1	130.97 (10)	C14—C13—H13	116.5
C1—N1—In1	117.67 (10)	C15—C14—C13	116.49 (15)
C6—N1—In1	124.12 (11)	C15—C14—C19	119.25 (15)
C6—N1—C1	117.74 (14)	C19—C14—C13	124.26 (14)
C2—N2—In1	116.64 (10)	C14—C15—H15	119.2
C13—N2—In1	125.01 (11)	C16—C15—C14	121.52 (16)
C13—N2—C2	118.19 (13)	C16—C15—H15	119.2
C3—N3—In1	116.55 (10)	C15—C16—H16	120.4
C20—N3—In1	125.81 (11)	C15—C16—C17	119.20 (15)
C20—N3—C3	117.44 (14)	C17—C16—H16	120.4
N1—C1—H1A	109.2	C16—C17—H17	119.7
N1—C1—H1B	109.2	C18—C17—C16	120.70 (16)
N1—C1—C4	112.22 (13)	C18—C17—H17	119.7
H1A—C1—H1B	107.9	C17—C18—H18	119.2
C4—C1—H1A	109.2	C17—C18—C19	121.68 (16)
C4—C1—H1B	109.2	C19—C18—H18	119.2
N2—C2—H2A	109.1	O2—C19—C14	123.92 (14)
N2—C2—H2B	109.1	O2—C19—C18	118.37 (14)
N2—C2—C4	112.62 (13)	C18—C19—C14	117.64 (14)
H2A—C2—H2B	107.8	N3—C20—H20	116.5
C4—C2—H2A	109.1	N3—C20—C21	126.97 (15)
C4—C2—H2B	109.1	C21—C20—H20	116.5
N3—C3—H3A	109.1	C22—C21—C20	115.34 (14)
N3—C3—H3B	109.1	C22—C21—C26	119.59 (14)
N3—C3—C4	112.59 (13)	C26—C21—C20	124.93 (14)
H3A—C3—H3B	107.8	C21—C22—H22	118.9
C4—C3—H3A	109.1	C23—C22—C21	122.16 (16)
C4—C3—H3B	109.1	C23—C22—H22	118.9
C1—C4—C2	111.33 (14)	C22—C23—H23	120.8
C3—C4—C1	110.68 (13)	C22—C23—C24	118.34 (16)
C3—C4—C2	111.51 (14)	C24—C23—H23	120.8
C5—C4—C1	108.12 (14)	C23—C24—H24	119.4
C5—C4—C2	107.57 (13)	C25—C24—C23	121.18 (15)
C5—C4—C3	107.45 (14)	C25—C24—H24	119.4
C4—C5—H5A	109.5	C24—C25—H25	119.2
C4—C5—H5B	109.5	C24—C25—C26	121.57 (15)
C4—C5—H5C	109.5	C26—C25—H25	119.2
H5A—C5—H5B	109.5	O3—C26—C21	124.64 (14)
H5A—C5—H5C	109.5	O3—C26—C25	118.23 (14)
H5B—C5—H5C	109.5	C25—C26—C21	117.11 (14)
N1—C6—H6	116.4	Cl1—C27—H27A	109.4
N1—C6—C7	127.16 (15)	Cl1—C27—H27B	109.4
C7—C6—H6	116.4	Cl2—C27—Cl1	111.12 (9)
C8—C7—C6	115.79 (15)	Cl2—C27—H27A	109.4
C8—C7—C12	119.30 (15)	Cl2—C27—H27B	109.4
C12—C7—C6	124.71 (14)	H27A—C27—H27B	108.0
			
In1—O1—C12—C7	18.4 (2)	C6—C7—C12—C11	−173.00 (15)
In1—O1—C12—C11	−164.25 (11)	C7—C8—C9—C10	1.0 (3)
In1—O2—C19—C14	31.0 (2)	C8—C7—C12—O1	179.02 (15)
In1—O2—C19—C18	−152.00 (11)	C8—C7—C12—C11	1.6 (2)
In1—O3—C26—C21	11.0 (2)	C8—C9—C10—C11	0.3 (3)
In1—O3—C26—C25	−170.70 (11)	C9—C10—C11—C12	−0.6 (3)
In1—N1—C1—C4	34.62 (17)	C10—C11—C12—O1	−177.97 (16)
In1—N1—C6—C7	−11.7 (2)	C10—C11—C12—C7	−0.4 (2)
In1—N2—C2—C4	36.43 (16)	C12—C7—C8—C9	−2.0 (2)
In1—N2—C13—C14	−4.6 (2)	C13—N2—C2—C4	−147.93 (15)
In1—N3—C3—C4	37.20 (17)	C13—C14—C15—C16	−179.20 (15)
In1—N3—C20—C21	1.6 (2)	C13—C14—C19—O2	−4.1 (2)
N1—C1—C4—C2	41.27 (18)	C13—C14—C19—C18	178.85 (15)
N1—C1—C4—C3	−83.35 (17)	C14—C15—C16—C17	0.0 (3)
N1—C1—C4—C5	159.22 (14)	C15—C14—C19—O2	175.61 (14)
N1—C6—C7—C8	178.28 (16)	C15—C14—C19—C18	−1.5 (2)
N1—C6—C7—C12	−6.9 (3)	C15—C16—C17—C18	−0.7 (3)
N2—C2—C4—C1	−84.71 (17)	C16—C17—C18—C19	0.3 (3)
N2—C2—C4—C3	39.44 (18)	C17—C18—C19—O2	−176.42 (15)
N2—C2—C4—C5	157.01 (14)	C17—C18—C19—C14	0.8 (2)
N2—C13—C14—C15	171.32 (16)	C19—C14—C15—C16	1.1 (2)
N2—C13—C14—C19	−9.0 (3)	C20—N3—C3—C4	−137.89 (15)
N3—C3—C4—C1	40.04 (19)	C20—C21—C22—C23	175.90 (15)
N3—C3—C4—C2	−84.47 (17)	C20—C21—C26—O3	4.7 (2)
N3—C3—C4—C5	157.89 (14)	C20—C21—C26—C25	−173.64 (14)
N3—C20—C21—C22	173.34 (16)	C21—C22—C23—C24	−1.6 (3)
N3—C20—C21—C26	−11.0 (3)	C22—C21—C26—O3	−179.78 (15)
C1—N1—C6—C7	176.28 (15)	C22—C21—C26—C25	1.9 (2)
C2—N2—C13—C14	−179.82 (15)	C22—C23—C24—C25	1.3 (3)
C3—N3—C20—C21	176.14 (15)	C23—C24—C25—C26	0.6 (3)
C6—N1—C1—C4	−152.81 (14)	C24—C25—C26—O3	179.35 (15)
C6—C7—C8—C9	173.12 (16)	C24—C25—C26—C21	−2.2 (2)
C6—C7—C12—O1	4.4 (3)	C26—C21—C22—C23	0.0 (2)

### ({[(2,2-Bis{[(2-oxidobenzylidene)amino-κ2N,O]methyl}propyl)imino]methyl}phenololato-κ2N,O)indium(III) dichloromethane monosolvate (2) Hydrogen-bond geometry (Å, º)

**Table:**

*D*—H···*A*	*D*—H	H···*A*	*D*···*A*	*D*—H···*A*
C6—H6···O2^i^	0.93	2.65	3.3596 (19)	134
C8—H8···O2^i^	0.93	2.63	3.394 (2)	139
C27—H27*B*···O1	0.97	2.26	3.193 (2)	160
C27—H27*B*···O2	0.97	2.82	3.253 (2)	108
C27—H27*B*···O3	0.97	2.73	3.411 (2)	127

Symmetry code: (i) *x*, −*y*+3/2, *z*+1/2.
